# Biotechnological production of omega-3 fatty acids: current status and future perspectives

**DOI:** 10.3389/fmicb.2023.1280296

**Published:** 2023-11-07

**Authors:** Jiansong Qin, Elif Kurt, Tyler LBassi, Lucas Sa, Dongming Xie

**Affiliations:** Department of Chemical Engineering, University of Massachusetts Lowell, Lowell, MA, United States

**Keywords:** omega-3 fatty acids, eicosapentaenoic acid, docosahexaenoic acid, biomanufacturing, alternative carbon sources, microalgae, yeast

## Abstract

Omega-3 fatty acids, including alpha-linolenic acids (ALA), eicosapentaenoic acid (EPA), and docosahexaenoic acid (DHA), have shown major health benefits, but the human body’s inability to synthesize them has led to the necessity of dietary intake of the products. The omega-3 fatty acid market has grown significantly, with a global market from an estimated USD 2.10 billion in 2020 to a predicted nearly USD 3.61 billion in 2028. However, obtaining a sufficient supply of high-quality and stable omega-3 fatty acids can be challenging. Currently, fish oil serves as the primary source of omega-3 fatty acids in the market, but it has several drawbacks, including high cost, inconsistent product quality, and major uncertainties in its sustainability and ecological impact. Other significant sources of omega-3 fatty acids include plants and microalgae fermentation, but they face similar challenges in reducing manufacturing costs and improving product quality and sustainability. With the advances in synthetic biology, biotechnological production of omega-3 fatty acids via engineered microbial cell factories still offers the best solution to provide a more stable, sustainable, and affordable source of omega-3 fatty acids by overcoming the major issues associated with conventional sources. This review summarizes the current status, key challenges, and future perspectives for the biotechnological production of major omega-3 fatty acids.

## 1. Introduction

**Figure 1:**
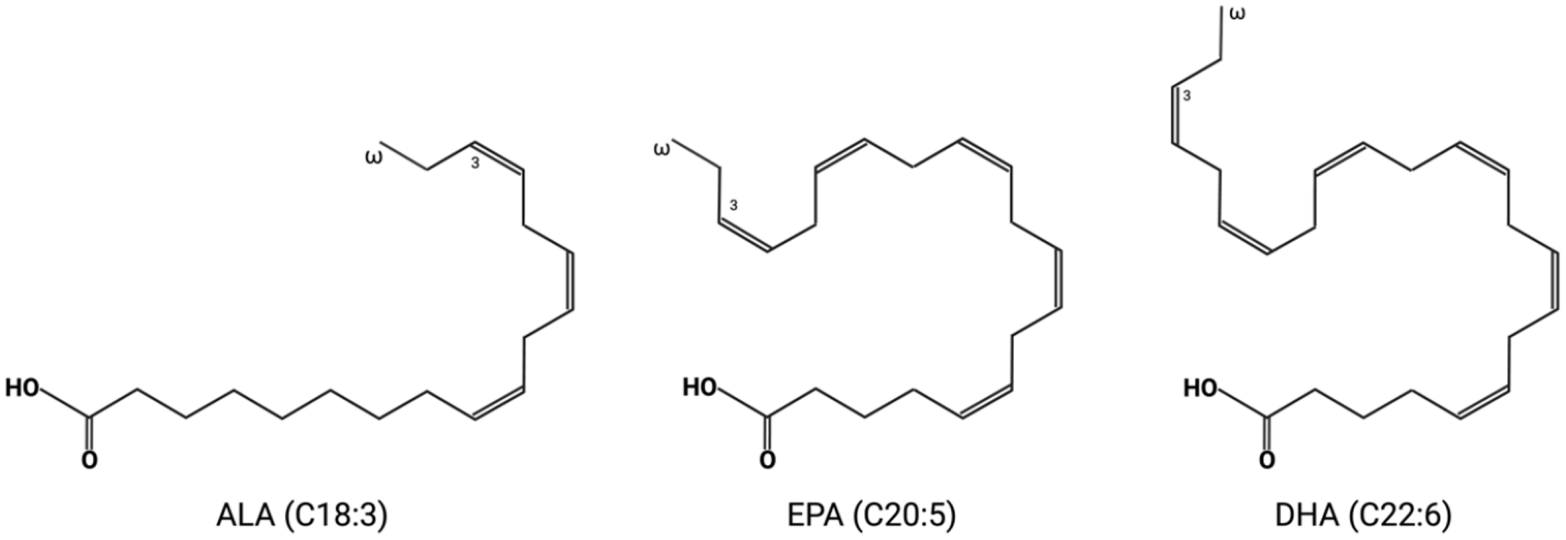
The molecular structure of three major omega-3 fatty acids: alpha-linolenic acid (ALA, C18:3), eicosapentaenoic acid (EPA, C20:5), and docosahexaenoic acid (DHA, C22:6).

Each fatty acid molecule has two ends: the methyl end (or omega end) and the carboxyl end (or alpha end). Omega-3 fatty acids are long-chain polyunsaturated fatty acids (LC-PUFAs) that have first double bonds at the third carbon from the omega end of the fatty acid chain. Major omega-3 fatty acids studied in this paper include alpha-linolenic acid (ALA, C18:3), eicosapentaenoic acid (EPA C20:5), and docosahexaenoic acid (DHA C22:6) (Shahidi and Ambigaipalan, 2018; Figure 1). The unique chemical structure of long carbon chains and multiple double bonds gives omega-3 fatty acids, especially the EPA and DHA, distinctive properties that may lead to significant health benefits. After esterification, EPA and DHA can insert into the membrane phospholipid bilayer, interact with the surrounding phospholipid, alter lipid rafts, influence the rate of oxidation and signal transduction pathway, and decrease cholesterol accumulation in the cell membrane (Hashimoto et al., 1999; Mason and Jacob, 2015; Mason et al., 2016; Sherratt et al., 2021).

As essential parts of the human cellular structure (Sherratt et al., 2021), omega-3 fatty acids are primarily found in the central nervous system, testes, heart, retina, and immune system (Hashimoto et al., 1999; Cholewski et al., 2018). There is solid evidence demonstrating that omega-3 fatty acids can be used as anti-cancer and anti-inflammation agents (Mason and Jacob, 2015; Mason et al., 2016), improve the cardiovascular, mental, and immune systems, and provide other health benefits in nerves, eyes, bones, and muscles (Molfino et al., 2014; Djuricic and Calder, 2021).

Yokoi-Shimizu et al. (2022) have shown that EPA and DHA can affect melatonin, a hormone crucial for sleep regulation. They can modulate melatonin production by modifying the pineal gland’s cell membrane structure, the organ tasked with producing this hormone. This alteration, in turn, influences human sleep patterns. Furthermore, evidence exists that both EPA and DHA can treat and even prevent anxiety and depression in adults. Therefore, the evidence strongly supports the idea that an increased intake of EPA and DHA can enhance both nervous and mental health (Kiecolt-Glaser et al., 2011; Appleton et al., 2015). EPA and DHA can also counteract the harmful impacts of muscle atrophy and hasten neuromuscular adaptation (Jeromson et al., 2015; Ochi and Tsuchiya, 2018). They can intervene in the signal transduction pathway in various cell types and thus play a preventive role in pathological calcification, like vascular calcification and microcalcification in cancer tissues. In parallel, these fatty acids improve bone quality by preventing bone decay and augmenting bone mineralization (Sharma and Mandal, 2020).

Consequently, EPA and DHA safeguard and enhance bone and muscle health. Regarding the visual system, omega-3 fatty acids may offer notable benefits as they have been shown to treat dry eye disease (Pellegrini et al., 2020) and help manage myopia effectively (Pan et al., 2021). It has also been reported that consumption of EPA and DHA can lower the risk of various types of cancer, including colon and breast cancer (Fabian et al., 2015; Augimeri and Bonofiglio, 2023). For cancer patients, omega-3 fatty acids can interact with G protein-coupled receptors GPR40/FFA1 and GPR120/FFA4 as agonists to alleviate cancer-related complications like paraneoplastic syndromes, pain, depression, and anorexia-cachexia syndrome (Freitas and Campos, 2019). Further studies showed that EPA and DHA can alter phosphate fatty acid distribution and lipid raft position, inhibit the inflammation transcript factor, and activate the anti-inflammation factor (Calder, 2017), allowing them to provide anti-inflammation benefits. Therefore, native Eskimos from Greenland and Japanese people who consume high amounts of omega-3 fatty acids from seafood have lower incidences of myocardial infarction and chronic inflammatory or autoimmune disorders (Simopoulos, 2002). In addition, it is believed that EPA and DHA can reduce cardiovascular disease risk in general. Research indicates that a daily intake of 2–4 g of combined EPA and DHA decreases cardiovascular events in individuals with cardiovascular disease (Elagizi et al., 2021). The consumption of these fatty acids can notably assist in mitigating cardiovascular ailments. Specifically, omega-3 fatty acids have been shown to reduce atherosclerotic cardiovascular disease and lower blood pressure in individuals with hypertension (Bercea et al., 2021; Patel and Busch, 2021).

However, due to the lack of delta-12 desaturase (D12Des), the human body is not able to synthesize linoleic acid (LA, C18:2) and alpha-linoleic acid (ALA, C18:3) from palmitic acid (PA, C16:0) and oleic acid (OLA, C16:1) (Lupette and Benning, 2020), and the pathway to synthesize EPA and DHA from LA or ALA is inefficient in humans. Therefore, the conversion rate from ALA to EPA and DHA is only 0.2–0.8% and < 4% in men, 21, and 9% in women, respectively (Childs et al., 2014). It is suggested that individuals include EPA and DHA in their daily dietary intake to promote better health, and the American Heart Association suggests that 4 g/day of EPA and DHA or EPA only be used as daily supplements (Siscovick et al., 2017; Skulas-Ray et al., 2019), which leads to a substantial demand for EPA and DHA on the market. Currently, the major source of omega-3 fatty acids is fish oil, which costs an average of 14 dollars/kg wholesale price. It is estimated that the global market for omega-3 fatty acids was 2.10 billion USD in 2020, and with an annual growth rate of 7.8%, it will reach 3.61 billion in 2028 (Benvenga et al., 2022).

Fish (mainly sardine fish) consume microalgae in ocean water (Wen and Chen, 2003), which leads to the accumulation of microalgae-produced omega-3 fatty acids in fish bodies (Calder, 1996). However, the sustainability of fish oil-based omega-3 fatty acids is in question due to overfishing, inconsistency of omega-3 contents, and potential contamination in the ocean (Kris-Etherton et al., 2002). Wild-type and engineered microalgae can also be used to produce omega-3 fatty acids. For example, Martek used microalgae to produce DHA as the major omega-3 ingredient for infant formula (Morrow, 2003; Spolaore et al., 2006; Arterburn et al., 2007). It is still challenging to produce EPA or both EPA and DHA at high yields using a microalgae-based fermentation process. Recently, DuPont has developed a land-based source of omega-3 by using yeast fermentation technology (Xie et al., 2015). Specifically, the yeast *Yarrowia lipolytica* was metabolically engineered to use sugars from agriculture feedstocks to produce omega-3 EPA via large-scale fermentation processes. The conversion yield from sugar(s) to the omega-3 fatty acids is still a major challenge for yeast fermentation to lower the manufacturing cost further. In addition to the efforts above, plants such as canola can also be engineered to produce omega-3 fatty acids in seeds, but it takes months to harvest and purify the omega-3 fatty acids from the plant oil (Abbadi et al., 2004; Venegas-Calerón et al., 2010; Chen et al., 2014). Overall, scientists have made significant progress in the biotechnological production of omega-3 fatty acids, but major challenges remain to overcome the current supply shortage to meet the increased demand for the product.

This review aims to provide a detailed insight into the current status of biotechnology production of omega-3 fatty acids and project future trends and opportunities in this rapidly evolving field. First, the current major sources and manufacturing technologies for omega-3 fatty acids are reviewed, and the main challenges are summarized. After that, the microbial production of omega-3 fatty acids and the main feedstocks or their alternatives for microbial production of omega-3 are discussed. To understand the potential new strategies for biotechnological production of omega-3 fatty acids, major metabolic engineering strategies for improving the yield of omega-3 fatty acids are reviewed, which include the principal molecular and synthetic biology tools employed to optimize fatty acid biosynthesis pathways in microbial hosts. Finally, future perspectives in new strain engineering and biomanufacturing strategies are suggested for high-yield, low-cost, and large-scale biomanufacturing of omega-3 fatty acids.

## 2. Current major sources for omega-3 fatty acids and their limitations

### 2.1. General metabolic pathways for biosynthesis of omega-3 fatty acids

**Figure 2:**
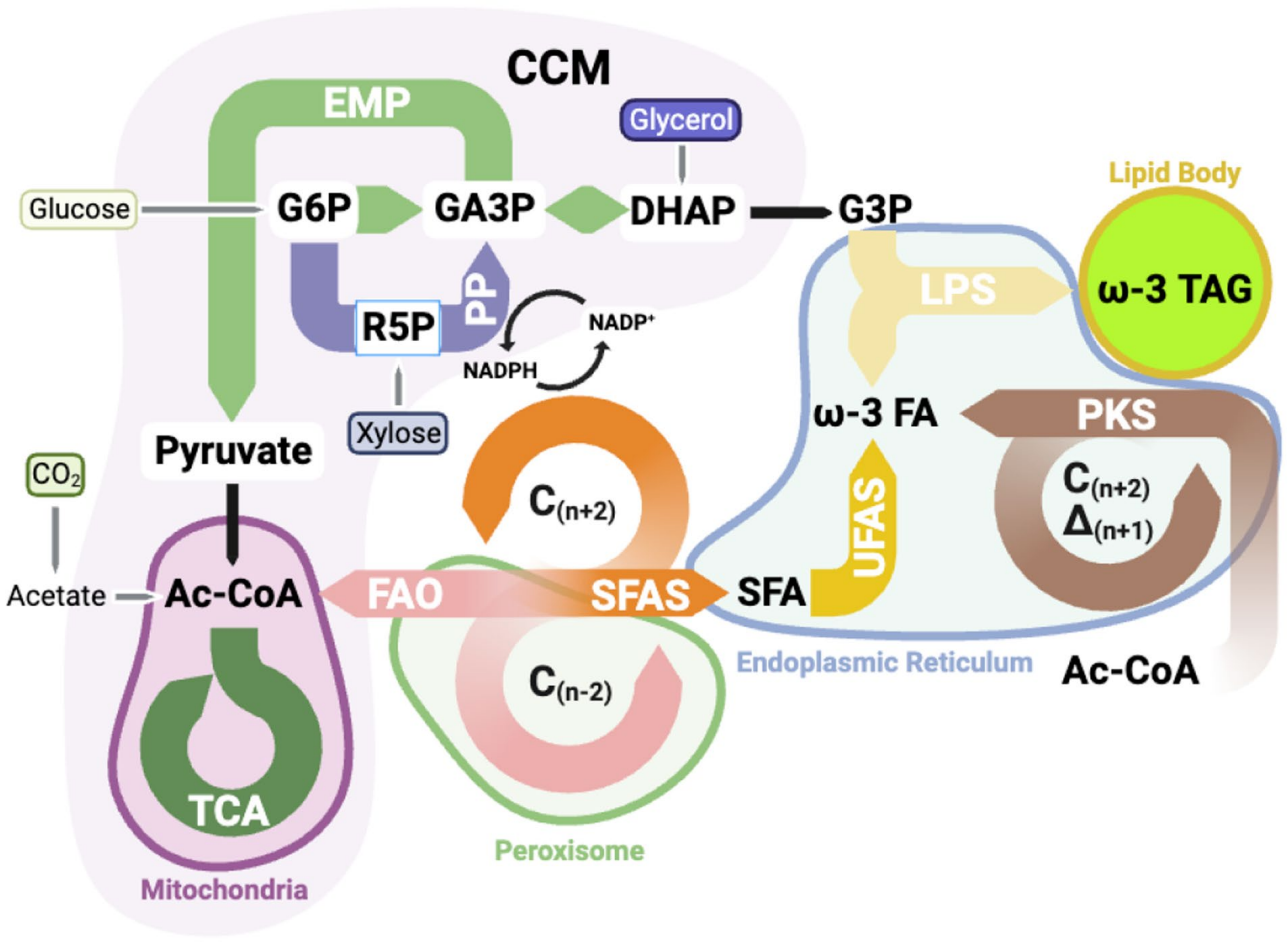
The metabolic pathway is for synthesizing omega-3 fatty acids, which consists of the Central Carbon Metabolic (CCM) pathway, fatty acid synthesis (FAS) pathway, and the lipid synthesis pathway (LPS). (1) The CCM pathway, including Glycolysis (EMP), Pentose Phosphate pathway (PP), and Tricarboxylic Acid Cycle (TCA), contributes the precursor for omega-3 fatty acids synthesis and converts various heterology organic carbon sources, such as glucose, acetate, and xylose glycerol…, into a building block, electro carrier, and energy for fatty acids synthesis. (2) The FAS pathway includes the Saturated Fatty Acids Synthesis (SFAS) pathway, the Unsaturated Fatty Acids Synthesis (UFAS) pathway, and an alternative polyketide synthesis (PKS) pathway. The acetyl coenzyme A (Ac-CoA) can be further converted to omega-3 fatty acids using the electro carrier (NADPH) generated from the PP pathway. However, the fatty acids can convert to Acetyl-CoA through the Fatty Acid Beta-Oxidation pathway (FAO). (3) The LPS pathway involves attaching all the omega-3 fatty acids to the glycerol backbone. G6P, Glucose 6-Phosphate; GA3P, Glyceraldehyde 3-Phosphate; R5P, Ribose 5-phosphate; DHAP, Dihydroxyacetone phosphate; G3P, Glycerol 3-phasphate. SFA, Saturated Fatty Acids; omega-3 FA, omega-3 fatty acids; omega-3 TAG, omega-3 triglyceride.

The general metabolic pathway map (Figure 2) from different carbon sources to omega-3 fatty acids (Geddes and Oresnik, 2014) helps better understand the biosynthesis of omega-3 fatty acids in various organisms.

The synthesis of omega-3 fatty acids involves multiple metabolic pathways, including:

(1) Central carbon metabolism (CCM), including:

(a) Glycolysis pathway (EMP)
(b) Pentose phosphate pathway (PP)
(c) Tricarboxylic acid cycle (TCA)

(2) Fatty acids beta-oxidation pathway (FAO)
(3) Saturated fatty acid synthesis pathway (SFAS)
(4) Long-chain unsaturated fatty acid synthesis pathway (UFAS)
(5) Lipids synthesis pathway (LPS) (Xie et al., 2016)

Each pathway works synergistically for omega-3 fatty acids synthesis. Some pathways provide crucial intermedia metabolites like NADPH and acetyl-CoA for omega-3 fatty acids synthesis, but some pathways may consume too much carbon to generate byproducts like ethanol, acetate, and citrate. Up-regulating the pathway synthesizing important intermediate metabolites can “pull” the carbon flow to the omega-3 fatty acids. Evidence shows that producing enough NADPH is the primary limiting step during the fatty acid synthesis processing (Qiao et al., 2017). So, increasing the amount of NADPH is crucial for lipid and omega-3 fatty acids accumulated inside the cells. Down-regulating by blocking the branch of the pathway toward the byproduct to “push” the carbon flow to the product. These modifications of metabolic pathways may increase the titer and productivity of omega-3 fatty acids biomanufacturing. Thus, understanding each pathway related to omega-3 fatty acids synthesis is essential for biomanufacturing.

Several carbon sources can directly or indirectly enter the omega-3 synthesis metabolic pathway for omega-3 fatty acids biosynthesis, such as glucose, glycerol, acetate, xylose, and CO_2_. Here is an example of the synthesis of omega-3 fatty acids from glucose. Glucose goes through the EMP and is degraded to glucose-6-phosphate (G6P) (Taymaz-Nikerel et al., 2018). While part of G6P, produced during glycolysis, enters the PP and converts the NADP^+^ to NADPH, the NADPH can provide the electron for fatty acid synthesis as an electron carrier (Xie et al., 2015). Afterward, under glycolysis, part of G6P is converted to acetyl-CoA. The acetyl-CoA can also be directed to the TCA cycle for energy generation within mitochondria, which is essential for cell survival and other activities (Xu, 2022).

Additionally, acetyl-CoA can enter the SFAS pathway, serving as the carbon source for synthesizing the carbon chain of omega-3 fatty acids. Once acetyl-CoA is converted into short-chain to medium-chain fatty acids through UFAS, these saturated fatty acids can enter the endoplasmic reticulum (ER) and be desaturated to form long-chain omega-3 fatty acids. It is important to note that these fatty acids can be degraded back to acetyl-CoA through FAO, which contains high energy and can serve as an energy source. Omega-3 fatty acids in the form of free fatty acids (FFA) need to be converted to the triglyceride format for cell storage through the lipid synthesis pathway (LPS).

Currently, the main source of omega-3 fatty acids is provided by fish oil. Other sources include the lipids/oils obtained from plants/vegetables, microalgae, fungi/yeast, and bacteria. Table 1 summarizes the current status of the major omega-3 fatty acid sources and their limitations in meeting the increased demand for omega-3 products.

**Table 1:** Biomanufacturing of omega-3 fatty acids from various organisms.

	Organisms	EPA		DHA		TFA/_DCW_	Gene Modification	References
		E_/TFA_	E/_DCW_	D/_TFA_	D/_DCW_			
Microalgae	*V. vischeri*	8.24	-	-	-	-	N	Yakoviichuk et al. (2023)
	*M. subterraneus*	32.9	3.8	-	-	11.6	N	Qiang et al. (1997)
	*N. oceanica*	61.9	2.3	-	-	3.8	N	Patil et al. (2007)
	*N. oculata*	18.7	-	-	-	-	N	Aussant et al. (2018)
	*Stauroneis* sp.	27.0	0.4	-	-	-	N	Archer et al. (2019)
	*Chlorella* sp.	-	-	-	0.7	-	N	Rasheed et al. (2020)
	*P. shiwhaense*	19.0	0.2	36.0	0.4	1.0	N	Jang et al. (2017)
	*G. smaydae*	8.4	0.4	43.0	2.1	4.9	N	Sivakumar et al. (2022)
	*Phaeothamnion* sp.	12.0	1.2	7.0	0.3	-	N	Archer et al. (2019)
	*Schizochytrium sp*	50	-	-	-	-	N	Lopes Da Silva et al. (2019)
	*Nannochloropsis salina*	16.3	3.5	-	-	-	N	Hoffmann et al. (2010)
	*Diacronema* sp.	2.0	0.2	4.0	0.2	-	N	Archer et al. (2019)
Fungi	*P. irregulare*	0.3	-	0.5	-	-	Y	O’brien et al. (1993)
	*Aurantiochytrium* sp.	10.4	-	40.2	-	-	Y	Li et al. (2015)
	*M. alpina*	26.4	-	-	-	-	Y	Okuda et al. (2015)
	*Y. lipolytica*	56.6	15.0	-	-	26.5	Y	Xue et al. (2013) and Xie et al. (2015)
	*Mortierella* sp.	6.7	-	4.9	-	-	Y	Vadivelan and Venkateswaran (2014)
	*M. alpina*	31.5	-	-	-	12.7	Y	Tang et al. (2018)
	*A. limacinum*	-	-	15.0	-	-	Y	Abad and Turon (2015)
	*S. limacinum*	-	-	15.8	-	-	Y	Chi et al. (2009)
	*C. guilliermondii*	2.8	-	6.7	-	-	Y	Guo and Ota (2000)
Bacteria	*Myxobacteria* spp.	10.3	-	11.9	-	-	Y	Garcia et al. (2016)
	*E. coli*	-	-	31.7	-	-	Y	Giner-Robles et al. (2018)
	*E. coli*	0.4	-	-	-	-	Y	Thiyagarajan et al. (2021)
	*L. lactis*	-	0.01	-	0.1	-	Y	Amiri-Jami et al. (2014)
Plants	*B. napus*	0.3–0.5	-	6.5–10.3	-	23.9–35.8	Y	MacIntosh et al. (2021)
	*C. sativa*	24.0	-	8.0	-	-	Y	Ruiz-Lopez et al. (2014)
	*A. hypogaea L.*	68.0	-	-	-	-	Y	Wang C. et al. (2019)
	*B. napus*	-	-	9–11	-	-	Y	Petrie et al. (2020)
	*Arabidopsis*	2.5	-	0.4	-	-	Y	Graham et al. (2007)
	*Brassica juncea*	15	-	1.5	-	-	Y	Wu et al. (2005)
	*Glycine max*	19.6	-	3.3	-	-	Y	Chen et al. (2014)
	*Brassica carinata*	25	-	-	-	-	Y	Cheng et al. (2010)
	*Brassica napus*	-	-	10	-	-	Y	MacIntosh et al. (2021)
	*Camelina sativa*	24	-	-	-	-	Y	Ruiz-Lopez et al. (2014)
	*Camelina sativa*	8	-	11	-	-	Y	Ruiz-Lopez et al. (2014)

E_/TFA_, EPA content in Total fatty acids (%); E_/DCW_, EPA content in Dry Cell Weight (%); D_/TFA_, DHA content in Total fatty acids (%); D_/DCW_, DHA content in Dey Cell Weight (%).

### 2.2. Omega-3 fatty acids from fish

Marine organisms, including fish, algae, shellfish, etc., usually contain relatively higher concentrations of omega-3 fatty acids than land animals or plants. For example, salmon contains 35.5 g of omega-3 fatty acid per 100 g of fish oil, but corn only contains 0.9 g per 100 g of corn oil (Rubio-Rodríguez et al., 2010). Although most marine animals, including fish, have limited omega-3 fatty acids biosynthesis ability, they can enrich omega-3 fatty acids from microalgae through the food chain (Wen and Chen, 2003). Therefore, marine animals at a relatively higher ecology niche can enrich omega-3 fatty acids at a relatively higher content by predating other creatures at a lower trophic level (Calder, 1996). Fish at a high trophic level have relatively high concentrations of omega-3 fatty acids in their body among all marine organisms, such as microalgae, shellfish, and squid. Moreover, fish is more accessible to pretreat than other organisms like shellfish, shrimp, and crabs with hard shells. Overall, fish oil extraction from fish is the leading source for humans to obtain omega-3 fatty acids from nature currently, especially for producing EPA and DHA products.

However, producing omega-3 fatty acids from fish may not necessarily be the best option due to several major limitations: (1) The omega-3 fatty acid content in fish fillet or oil is influenced by a wide range of factors, including fish species, the diet of the fish, environmental temperature, age and sexual maturity of the fish, part of the fish processed, health of the fish, season, geometrical location and weather, and farming or wild-caught (Strandberg et al., 2020; Alfio et al., 2021). (2) Ocean and lake contamination affects the safety of omega-3 fatty acids in fish (Domingo, 2007; Tocher, 2009). Easton’s studies revealed that methylmercury and polychlorinated biphenyls were detected in four kinds of wild-caught or farm-raised salmon (Easton et al., 2002). Mercury in fish could negate cardioprotective effects, and exposure to other organic pollutants remains a concern for human health (Domingo et al., 2007). (3) Vitamins A and D extracted with omega-3 fatty acids from fish oil are another concern. It was suggested that excessive intake of vitamins A and D may be associated with other related diseases, such as elevated cholesterol and other saturated fatty acid absorption (Dave and Routray, 2018). Additionally, the absolute omega-3 fatty acids content in fish (2.51 g/100 g in salmon) (Shahidi and Ambigaipalan, 2018) is low and is a barrier to lowering the cost of industry-scale manufacturing.

Three main steps are required to extract omega-3 fatty acids from fish: (1) processing raw material (fish or fish waste), (2) extraction from processed raw material, and (3) refining of the extracted oil. Despite over 200 years of technological development, the current manufacturing cost remains high (Rubio-Rodríguez et al., 2010). While the demand for fish-based omega-3 fatty acids increases continuously, overfishing may become another concern (Lenihan-Geels et al., 2013), which may disrupt marine ecosystems, as evidenced by Frank et al.’s research on the Atlantic shelf ecosystem (Frank et al., 2005; Scheffer et al., 2005). Consequently, However, the regulation and limitation and the imbalance between demand and supply lead to a further increase in the production cost omega-3 from the fish (Oliver et al., 2020). The omega-3 fatty acids manufactured from fish are also not desired by vegetarians. Considering the health requirement of vegetarians, the omega-3 fatty acids market for vegetarians is growing, notable, and substantial (Leahy et al., 2010).

### 2.3. Omega-3 fatty acids from plants

As an essential part of agriculture, with more than thousands of years of development, growing plants on land are stable and with consistent yields. Producing omega-3 fatty acids by land plants is a possible alternative to fish production. Although plants have a limited metabolic pathway to synthesize omega-3 fatty acids, like EPA and DHA, with the development of molecular biotechnology, integrating the hetero omega-3 fatty acids synthesis pathway from microalgae, yeast, or bacteria to plants to produce omega-3 is more accessible (Adarme-Vega et al., 2014).

While plants can be the primary food source and synthesize oil efficiently, they natively lack the genes necessary for synthesizing the EPA and/or DHA fatty acids. Transgenic plants, which have been genetically modified, could potentially provide the backbone for omega-3 fatty acids production. Several research groups that transformed the completed biosynthesis pathway of aerobic fatty acid desaturation/elongation pathway to several land plants increased their omega-3 fatty acids content.

(1) *Arabidopsis* (Mouse-ear cress), after hetero-gene integration, the EPA and DHA content improved to 2.5 and 0.4%, respectively (Graham et al., 2007).
(2) *Brassica juncea* (Indian mustard) reached 15% EPA and 1.5% DHA after modification (Wu et al., 2005).
(3) Transgene soybean can accumulate 19.6% EPA and 3.3% DHA (Kinney et al., 2004; Chen et al., 2014).
(4) *Brassica carinata,* after inserting three different desaturases and two elongases for omega-3 synthesis, successfully produced seeds with a composition of 25% EPA (Cheng et al., 2010).
(5) Expressing seven genes on a vector control under seed-specific promoters in *Brassica napus* generated one of the first land-based DHA production systems. This transgenic DHA canola seed contains 10% DHA (MacIntosh et al., 2021).
(6) After gene modification in *Camelina sativa*, One iteration for EPA production can accumulate 24% EPA in the seed; another iteration for DHA and EPA production can accumulate 8% DHA and 11% EPA in the seed (Ruiz-Lopez et al., 2014).

The molecular biology technology used on plants makes the plants successfully produce omega-3 fatty acids.

However, plants have a nonnegligible bottleneck: the elongation and desaturation of the fatty acid happen in a substrate in a parallel format. The desaturase prefers the acyl-PC, and the elongase prefers the acyl-CoA (Domergue et al., 2003). Acyl-PC and acyl-CoA each have their pools for synthesizing omega-3 fatty acids. Given the different preferences of desaturase and elongase enzymes, there is a requirement for the efficient transfer from acyl-PC to acyl-CoA. This transfer is an additional step in the synthesis of omega-3 fatty acids. Therefore, these characteristics of plants contribute to their inefficiency in synthesizing omega-3 fatty acids. Another bottleneck is that after the synthesis, omega-3 fatty acids are in acyl-PC and acyl-CoA formats. These two acyl formats use different enzymes to synthesize triglycerides. One synthesis pathway is through the Kennedy pathway, while the other involves catalyzation by enzymes such as LPCAT and PDAT. Ensuring both pathways are efficient is vital for triglyceride synthesis. However, this means the process might not be as efficient as in other microbes, which primarily use one pathway for lipid synthesis (Abbadi et al., 2004; Chen et al., 2014).

### 2.4. Omega-3 fatty acids from microalgae

Microalgae are unicellular species containing eukaryotes and prokaryotes (Wen and Chen, 2003). The smallest microalgae are only a few microns, while the larger ones can reach a hundred microns and are widely distributed in the ocean and freshwater (Ryckebosch et al., 2012). As the only creature that can *de novo* synthesize omega-3 fatty acids efficiently in nature, historically, humans have commercially used microalgae for a long time as food, fodder, and a chemical of high value. Early Chinese references from 2,700 BC highlight the health benefits of microalgae for humans (Patil et al., 2005). Microalgae exhibit a faster growth rate than animals and plants, which are considered the conventional sources of omega-3 fatty acids (Perdana et al., 2021). They contain high lipid levels, with total lipids content per dry cell weight between 20–70% (Lowrey et al., 2016; Sun et al., 2017). In addition, the omega-3 fatty acids from microalgae are cholesterol-free, contamination-free, and fishy-odorless (Mendes et al., 2009). These properties make microalgae a potential alternative omega-3 fatty acid manufacturing source. Microalgae are adaptable microorganisms that can grow in phototrophic, heterotrophic, and mixotrophic (Barta et al., 2021), differentiating them using light, inorganic, and organic carbon sources. Photoautotrophic processing involves the *de novo* generation of omega-3 fatty acids from inorganic carbon sources (CO_2_) using light as an energy source through photosynthesis. This processing with a low carbon footprint is sustainable and cost-effective for omega-3 fatty acids production, but the light requirement leads to a high requirement of the photobioreactor design, which limits its development. Open ponds could serve as ideal environments for phototrophic microalgae. However, they are susceptible to environmental fluctuations and are costly to start (Russo et al., 2021).

Conversely, the heterotrophic process uses organic carbon sources, such as glucose, xylose, acetate, wastewater, and crop flour, to produce omega-3 fatty acids. All these inexpensive organic carbon sources can be used to manufacture omega-3 fatty acids through high-cell-density fermentation in stainless steel vessels without light. The high cell density will decrease the cost of producing omega-3 fatty acids (Winwood, 2013).

#### 2.4.1. Lipid and omega-3 synthesis in microalgae

As an alternative source of biomanufacturing omega-3 fatty acids to fish oil, microalgae offer a prospective future in non-polluted, sustainable, arable, land-free, and fast-growing operations (Couto et al., 2021). Microalgae are the initial omega-3 producers in the marine food chain and can grow faster under different trophic cultivation conditions (Adarme-Vega et al., 2012; Chen et al., 2023). Due to their fast-doubling time, for omega-3 fatty acids biomanufacturing, harvesting microalgae every 4–6 days is a typical turnover time, but it can only harvest land plants 2–3 times annually (Rittmann, 2008). Consequently, the accumulation of omega-3 fatty acids in microalgae and their high turnover rate make them a highly efficient and sustainable source of omega-3 fatty acids biosynthesis (Barone et al., 2020). Easy harvesting is another unique advantage of microalgae (Abidizadegan et al., 2021). As unicellular creatures, microalgae can accumulate omega-3 fatty acids evenly in each of their single cells. However, the concentration of omega-3 fatty acids is usually concentrated in some specific tissues or organisms of animals or plants. For instance, a high concentration of omega-3 fatty acids can be found in fish liver and plant seeds. Extra steps to separate specific tissues of animals or plants for omega-3 fatty acids harvesting may increase the manufacturing cost and generate unnecessary waste. This feature makes microalgae a more favorable source for omega-3 fatty acids product harvesting than plants or animals. The global food crisis is a critical topic. The production of omega-3 fatty acids from land plants relies on arable land, which potentially competes with food production.

Nevertheless, microalgae cultivation offers a promising alternative to circumvent these challenges (Rittmann, 2008). The aquatic environments for microalgae cultivation often provide more stable and consistent conditions than terrestrial ecosystems. Thus, the growth of microalgae and omega-3 fatty acid production is influenced less by seasonal and climate variation (Barta et al., 2021), ensuring a reliable production of omega-3 fatty acids. Furthermore, certain heterotrophic microalgae species can utilize sustainable, renewable carbon sources as substrate, saving the cost for omega-3 fatty acids biosynthesis and contributing to carbon balance. As a result, microalgae represent a potentially more consistent and environmentally friendly source for omega-3 fatty acids biomanufacturing.

Lipids accumulate inside microalgae cells or cell membranes under severe conditions against environmental change, especially the accumulation of omega-3 fatty acids. For example, omega-3 fatty acids will increase when the nutrients, such as nitrogen and phosphorus but light (Ramesh Kumar et al., 2019), are limited (Adarme-Vega et al., 2012). The accumulation of omega-3 fatty acids is not only because of their high energy but also because of their unique ability, such as good flow and antioxidizing ability, for cellular membrane function (Cohen et al., 2000). Because of the excellent fluid ability of omega-3 fatty acids, increasing omega-3 fatty acids content in cell membranes can maintain the fluidity of the membrane at low temperatures (Tatsuzawa and Takizawa, 1995; Jiang and Gao, 2004; Aussant et al., 2018). In some harsh conditions, like exposure to UV, which will generate free radicals and damage the membrane, the omega-3 fatty acids show great anti-oxidization ability to repair the cell membrane, consequently increasing the omega-3 fatty acids content in the cell membrane (Liang et al., 2006). These features that can lead to omega-3 fatty acid accumulation can be utilized for omega-3 fatty acid production. For example, purposely applying pressure to cultivate *Nannochloropsis salina* by decreasing the culture temperature tends to synthesize more EPA (Increased 40% EPA/dry cell weight) (Hoffmann et al., 2010).

Nevertheless, stress can induce the accumulation of omega-3 fatty acids; meanwhile, it also reduces growth rate and biomass, which leads to low lipid productivity (Ramesh Kumar et al., 2019) Thus, two-stage cultivation is considered a solution for this controversial scenario. The first stage is cultivation to increase biomass, then apply pressure in the second stage to produce omega-3 fatty acids (Lu et al., 2021).

Microalgae exhibit various trophic behaviors, including photoautotrophic, heterotrophic, and mixotrophic modes. Each mode presents unique advantages and disadvantages when biomanufacturing omega-3 fatty acids.

#### 2.4.2. Phototrophic cultivation

Most high EPA-content microalgae in nature thrive under a phototrophic mode. In the phototrophic mode, microalgae utilize the inorganic carbon source, CO2, and light as an energy source to photosynthesize carbohydrates. These carbohydrates subsequently enter the central carbon metabolic pathway, generating energy necessary for cellular survival. The energy derived from carbohydrates and the carbohydrates themselves are involved in synthesizing the biomass and omega-3 fatty acids (Barbosa et al., 2023).

Open systems (open ponds) and closed systems (photobioreactors, PBR) are applied in most of the microalgae phototrophic cultivation on an industry scale (Xia et al., 2020; Magoni et al., 2022). Most commercial microalgae cultivation is in the phototrophic mode in an open system. For example, several microalgae, such as *Scenedesmus* sp., *Chlorella* sp., and *Dunaliella* sp., are well-established commercially cultivated in ponds through autotrophic mode (Borowitzka, 1999). The major open-air systems include big shallow ponds, tanks, circular ponds, and raceway ponds, which directly utilize sunlight, leading to a more remarkable net energy ratio than a closed system (Jorquera et al., 2010). Thus, open ponds have significant economic efficiency and relatively lower operational costs (Sivakumar et al., 2022). Meanwhile, raceway systems are preferred in areas with high land costs to maximize cell density and minimize pond area (Borowitzka, 1999).

Open systems for microalgae come with their challenges. The unstable growth conditions of the open system are due to temperature and light exposure fluctuations. Additionally, the risk of contamination is higher in open systems, affecting both the microalgae’s development and the final product’s quality. Open-air systems often compromise between light availability, CO2 availability, and the need to maintain water depth for mixing and avoiding ionic composition changes due to evaporation (Borowitzka, 1999).

Nevertheless, a closed system can conquer part of the challenges in the phototrophic cultivation open system, even though the cost may be higher than the open system. Due to the enclosed system, the PBR system can control environmental parameters like temperature, nutrition concentration, and salinity, reduce contamination chances, and generate higher biomass (Santin et al., 2022). Also, they enable the cultivation of a more comprehensive range of species and operation over a broader climatic range. They allow for better control over culture conditions, ensuring consistent product quality. These systems can also operate in continuous culture mode, reducing harvesting costs and land requirements. However, the volume-to-surface ratio of PBR is limited, which restricts the lighting requirements when cultivating microalgae and consequentially restricts the scale-up (Martins et al., 2013). Meanwhile, oxygen will be the by-product of photosynthesis when phototrophic macroalgae cultivation. The accumulation of high oxygen in PBR affects the titers of biomass and omega-3 fatty acids (Mendes et al., 2009).

#### 2.4.3. Heterotrophic cultivation

Although phototrophic growing is more energy efficient than heterotrophic, it lowers the titer of biomass and lipids and the productivity of biomass and lipids (Davis et al., 2021). For instance, *Chlorella vulgaris* demonstrates higher biomass and lipids productivity under heterotrophic conditions than phototrophic conditions (Barone et al., 2020; Couto et al., 2021). In phototrophic cultivation, the maximum cell density reached so far has been observed in the cultivation of *M. salina* in thin-layer cascade photobioreactors, resulting in a dry cell weight of only 30 g L^−1^ dry cell weight and biomass productivity of 3.1 g L^−1^ D^−1^ (Schädler et al., 2020). In contrast, the highest reported cell density in heterotrophic cultivation has been achieved with *S. acuminatus* grown in a 7.5-L batch fermenter, producing a dry cell weight of 286 g L − 1 dry cell weight and biomass productivity of 91.4 g L^−1^ D^−1^ (Jin et al., 2020).

Certain microalgae demonstrate the remarkable ability to use sustainable and low-cost organic carbon sources as their carbon sources, thus allowing them to thrive in dark environments (Lopes Da Silva et al., 2019). This approach presents a distinct advantage over light-dependent phototrophic methods, as it is not restricted by the daily light cycle or geographical location. Moreover, it enables the growth of specific algal species that might not otherwise flourish in traditional phototrophic conditions (Saini et al., 2021). The growth under heterotrophic conditions employs fermenters, a well-understood technology extensively applied in various industries. As a result, this familiarity offers cost advantages as it reduces the complexity and financial burden associated with the design, operation, and subsequent scaling-up of production facilities. In addition to these benefits, the high productivity achieved in heterotrophic cultivation significantly shortens the culture time (Karageorgou et al., 2023). It also increases cell density cultures, greatly simplifying harvesting and downstream purification processes (Morales-Sánchez et al., 2013). A remarkable aspect of this approach is the continuous, day-and-night production of omega-3 fatty acids due to the independence from light, resulting in more consistent and predictable production. For instance, DSM, a global enterprise that acquired Martek Biosciences Corporation in 2010, leads the global production of DHA derived from algae. The company employs a heterotrophic microorganism, *Schizochytrium* sp., to produce algal oils marketed under Life’sDHATM and Life’sTM OMEGA. Remarkably, these algal oils contain a substantial 50% composition of EPA/DHA (Lopes Da Silva et al., 2019).

### 2.5. Omega-3 fatty acids from fungi

Using fungi as an omega-3 fatty acids production platform offers distinct advantages. Fungi can typically grow on several different carbon sources, making it a flexible choice for omega-3 fatty acids production. This multi-carbon source option allows the use of cost-effective resources like acetate, xylose, and glycerol. Furthermore, fungi can undergo high-density cell fermentation for omega-3 biomanufacturing, which has a higher titer of omega-3 fatty acids in a single bioreactor. This process saves a considerable amount of space and volume for fermentation. Fungi also have relatively high native lipid synthesis abilities, making them an ideal candidate for omega-3 fatty acids synthesis. For instance, *Yarrowia lipolytica*, engineered by DuPont, can produce EPA, comprising 50% of total lipids (Xue et al., 2013; Xie et al., 2015).

Three standards are necessary to value the cost efficiency of synthesizing omega-3 fatty acids: the dry cell weight of the microbe, the lipid content, and the omega-3 fatty acid content within the lipids (Zhang X.-Y. et al., 2022). The cells must synthesize and store the omega-3 fatty acids before harvesting and extraction. The dry cell weight determines the maximum lipid storage capacity; the lipid content dictates the maximum omega-3 synthesis potential; and the omega-3 fatty acids within the lipids represent the efficiency of omega-3 fatty acid synthesis. Oleaginous fungi, which can accumulate more than 20% of lipids within their cells (Patel et al., 2020), offer an alternative microbe type for consideration as a potential cell factory for omega-3 fatty acid synthesis. A recent research paper, represented in Table 1, reports the omega-3 fatty acids produced by fungi. Table 1 shows the omega-3 fatty acids produced by fungi in a recent research paper.

However, current omega-3 fatty acids manufacturing by fungi still faces major challenges and limitations, several of which are listed below:

(1) Compared to bacteria ( $\mu_{\text{max}}\sim 2$ h^−1^), fungi have a relatively low growth rate ( $\mu_{\text{max}}\leq 0.2\sim 0.3$ h^−1^), leading to a relatively lower productivity due to the lower growth rate (Lynch and Harper, 1974; Allen and Waclaw, 2019)
(2) Harvesting and purifying omega-3 fatty acids from oleaginous fungi present challenges limiting their development. The impurity of hydrophobic byproducts, such as omega-6 fatty acids, increases the difficulty of purification. The current primary purification method involves transesterifying fatty acids from freeze-dried cells, an energy-inefficient and costly process. Moreover, the harsh conditions may degrade the omega-3 fatty acids during processing. The product of this method can only be fatty acid methyl ester (FAME). The variability in cell membranes can also reduce the efficiency of this method (Gorte et al., 2020).
(3) In a typical scenario, aerobic fermentation necessitates a high oxygen uptake rate (OUR). Concurrently, high cell density and aerobic fermentation demand an elevated OUR. However, this requirement might exceed the capacity of most commercial-scale fermenters in terms of oxygen transfer rate (OTR) or cooling capacity. The latter is particularly important for temperature maintenance, as excessive heat generation could disrupt the process (Xie et al., 2017b).
(4) The low yield of lipids significantly limits the potential for industrial biomanufacturing of omega-3 fatty acids from fungi. *Y. lipolytica*, as an Oleaginous fungus, for example, can only theoretically yield 0.271 g of stearidonic acid (SA) per g of glucose. This limitation arises primarily because the synthesis of SA requires a sufficient supply of ATP and NADPH energy cofactors from the anabolic of glucose (Figure 2). Even if a metabolic engineering method can convert all NADH to NADPH, the theoretical yield only increases to 0.351 g per g of glucose (Qiao et al., 2017). Also, citric acid is usually produced as a byproduct during lipids synthesis, consuming 35% carbon source, which is a nonnegligible number (Liu et al., 2021). Moreover, more than 50% of carbon is lost as CO2 due to the need for ATP generation.
(5) Utilizing glucose as the primary carbon source has significant limitations. The sourcing of glucose, often derived from terrestrial plants, can compete with the demand for arable land for food crop production, potentially exacerbating global food crises. Additionally, converting glucose to omega-3 fatty acids is lengthy and inefficient, involving more than 20 steps. The low conversion yield, low productivity, and high capital costs associated with the batch or fed-batch process hinder the advancement of biomanufacturing omega-3 fatty acids from fungi. The product purification process, which involves the extraction of EPA/DHA from yeast biomass, is complex and costly. It necessitates a series of operations, including biomass harvesting by centrifugation and filtration, biomass drying, biomass disruption techniques such as extrusion, lipid extraction using hexane solvents, and distillation of DHA/EPA from the extracted lipids. These complexities and costs further increase the production cost of omega-3 fatty acids from fungi, decreasing its competitiveness. The new opportunities for omega-3 fatty acid biomanufacturing using fungi (Vasconcelos et al., 2018).

### 2.6. Omega-3 fatty acids from bacteria

Bacteria also present a viable host for biosynthesis of omega-3 fatty acids synthesis. Even though bacteria lack a native pathway for synthesizing omega-3 fatty acids, the molecular biology tools for engineering bacteria strains are mature, making it potentially easier to explore and develop more efficient omega-3 fatty acids synthesis pathways (Giner-Robles et al., 2018). Additionally, bacteria grow quickly, suggesting the potential for a shorter culture time to obtain omega-3 fatty acids products. However, bacteria face difficulty accumulating enough lipids within their cells, leading to low efficiency in producing omega-3 fatty acids.

The gene cluster for EPA/DHA expression was expressed in *E. coli* after removing redundant genes. It was demonstrated that EPA increased 3.5 to 6.1-fold. The optimized EPA/DHA expression cluster was inserted into *L. lactic* produced DHA at 0.135% of dry cell weight and EPA at 0.05% of dry cell weight (Amiri-Jami et al., 2014). Successfully producing EPA/DHA from *L. lactis* provides a faster and safer platform for EPA/DHA production. Although these bacterial hosts grow faster and can produce EPA and DHA, the relatively low yield prevents them from being ideal hosts for commercial omega-3 fatty acid manufacturing.

Presently, the production of omega-3 fatty acids by other bacteria, as detailed in Table 1, suffers from similar limitations. Owing to their low capability to accumulate lipids within cells and inefficiency in metabolizing lipids, the yield and titer of omega-3 fatty acids produced by bacteria are unsatisfactory. While there have been reports of bacteria-based production of omega-3 fatty acids, the resulting yield and concentration have not met acceptable standards.

### 2.7. Advantage and disadvantage of various organism

Based on the organisms, including microalgae, plants, fungi, and bacteria, discussed in this review, Table 2 summarizes the advantages and disadvantages of different organisms for omega-3 fatty acid production.

**Table 2:** Comparison between biomanufacturing of omega-3 fatty acids from various organisms.

	General information				Specific information	
	Growth rate	Omega-3 content	Gene editing	Space-cost	Advantage	Disadvantage
Plants	Very low	High	Hard	Very high	Can accumulate substantial lipids. Great potential for omega-3 production post-gene-editing. Phototrophic organisms capable of recycling CO_2_ back into omega-3 production. Environmentally friendly and cost-effective process.	Grow very slowly. Require large lands, Competes with food. High extraction cost due to the production of omega-3 in seeds. Major bottleneck in the native metabolic pathway for further improving omega-3 production. Relatively hard to engineer the plants.
Microalgae	Low	High	Easy	High	Can natively produce omega-3 fatty acids. Can accumulate high levels of omega-3. Mature technologies are available to cultivate them effectively. Relatively easy to conduct gene editing. The unicellular structure facilitates easier extraction of omega-3 fatty acids.	Low cell density. A large area is required for omega-3 production by phototrophic process. Light is necessary for phototrophic microalgae, necessitating a well-designed bioreactor with a higher surface area to accept light.
Fungi	High	Very high	Easy	Low	Grow rapidly in bioreactors and achieve very high cell density Require less space for industrial production. Easy to conduct gene editing. Oleaginous fungi can store high levels of lipid bodies. Capable of high omega-3 fatty acids production inside cells.	Grow relatively slower than bacteria. Substantial amounts of omega-6 fatty acids as by-products in fungi may increase the cost of purification processing. Require a large amount of oxygen during fermentation processing. Relatively low yield of omega-3 from glucose.
Bacteria	Very high	Low	Easy	Low	Fastest growth rate among all organisms. Most mature gene editing technology is available. Do not require too much space for industrial manufacturing.	Have shallow omega-3 content inside the cells due to the absence of an organelle structure that can accumulate sufficient lipids for omega-3 fatty acid production.

## 3. Metabolic pathways and engineering strategies for biosynthesis of omega-3 fatty acids

One key strategy to enhance the performance of microbial factories is by expanding the pathways that contribute to product synthesis and blocking those that may waste carbon, thereby directing carbon flow toward the desired product. Also, it is essential to ensure that energy blocks, such as ATP, NADH, and NADPH, balance with the energy required for product synthesis. Advancements in modern molecular biology make precise and delicate engineering of metabolic pathways within cells feasible. Among these tools, CRISPR is the most notable one. It can edit genes through targeted insertion, targeted knockout, gene expression activation, and gene expression inhibition. Protein engineering can also enhance an enzyme’s efficiency, directing reactions toward the desired outcome. These efficient molecular biology tools make the design of metabolic pathways more rational and productive.

### 3.1. Fatty acid synthesis and metabolism

#### 3.1.1. Fatty acid synthesis

**Figure 3:**
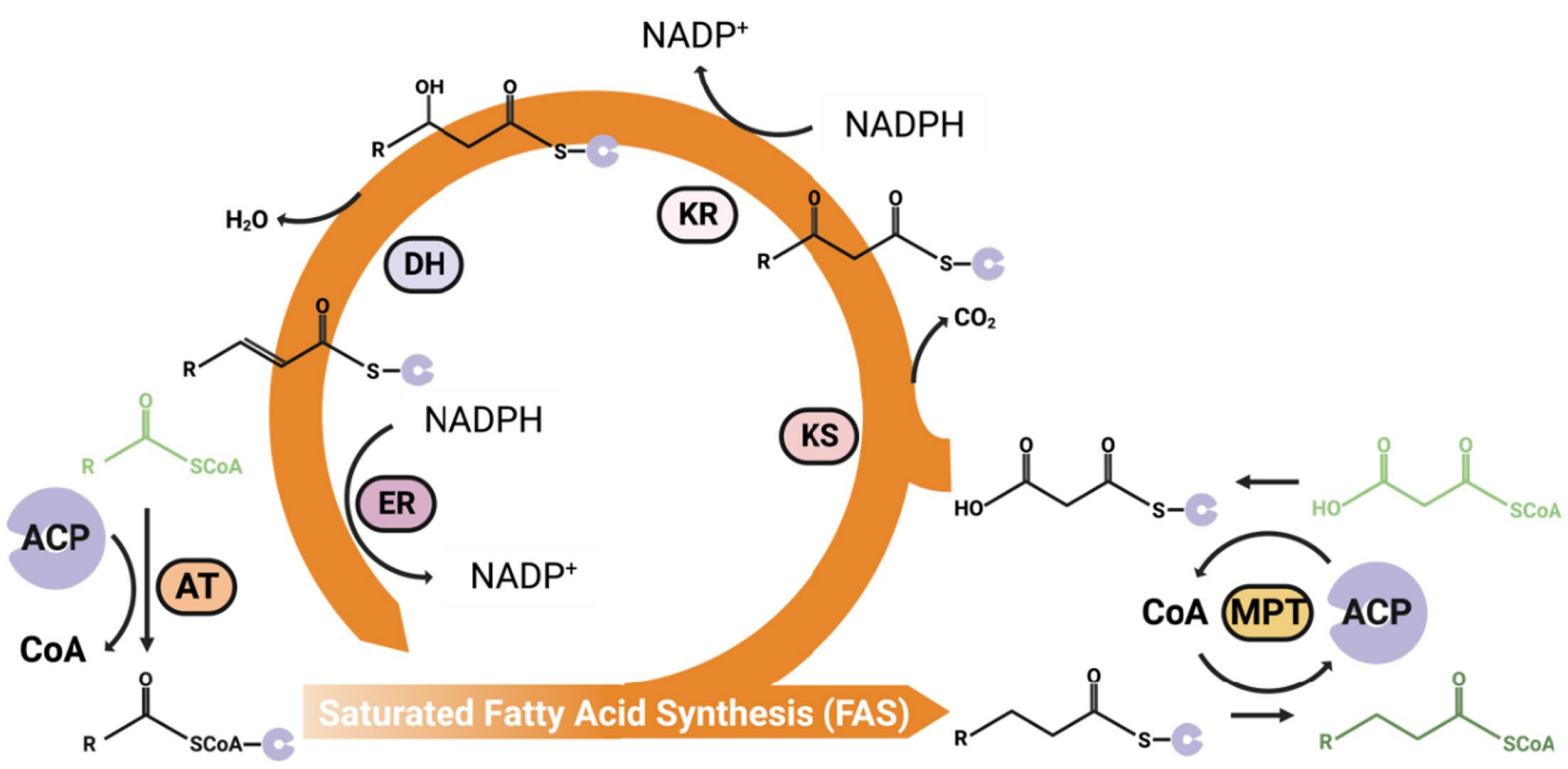
An overview of the general fatty acid synthesis process. The malonyl-CoA can add to acetyl-CoA every cycle of the FAS pathway to increase two carbons on the Acyl-CoA carbon chain. ACP, Acyl Carrier Protein; AT, Acetyltransferase; MPT, Malonyl/Palmitoyl Transferase; KS, Ketoacyl Synthase; KR, NADPH-dependent ketoacyl Reductase (KR); DH, dehydratase; ER, NADPH/FMN-dependent Enoyl Reductase.

The synthesis of fatty acids starts with acetyl-CoA, with each cycle adding two carbons to the fatty acid chain through malonyl-CoA, eventually synthesis of long-chain saturated fatty acids (Palmitic acids, C16:0) after multiple cycles, which mainly happened in the cytoplasm (Figure 3).

The yeast Fatty Acid Synthase (FAS) is a multi-enzymatic complex containing all necessary catalytic domains for producing a fully saturated C16 fatty acid from acetyl-CoA, malonyl-CoA, and NADPH. These domains, including acetyltransferase (AT), NADPH/FMN-dependent enoyl reductase (ER), dehydratase (DH), malonyl/palmitoyl transferase (MPT), NADPH-dependent ketoacyl reductase (KR), and ketoacyl synthase (KS), are distributed across two polypeptide chains. The cyclical fatty acid elongation process starts with AT transferring an acetyl group from acetyl-CoA to the Acyl Carrier Protein (ACP). A malonyl group from malonyl-CoA is then added to the ACP by the MPT. Then, the subsequent stages, occurring while intermediates remain attached to the ACP, involve:

(1) Combining malonyl-ACP with the acetyl primer that is attached to the KS to form acetoacetyl-ACP in an irreversible reaction.
(2) Reduction by KR.
(3) Dehydration by DH.
(4) Another reduction phase by ER.

The saturated fatty acid, now elongated by two carbon atoms, is transferred back to KS for the next cycle. Adding two carbons to the carbon chain even times, The MPT transfers the C_16_ (palmitoyl) from ACP to CoA and releases it to the cytoplasm (Maier et al., 2010). The fatty acid synthesis pathway requires substantial amounts of NADPH, as every two-carbon added to the carbon chain needs two NADPH molecules (Wu S. G. et al., 2016).

#### 3.1.2. General fatty acid degradation pathways

**Figure 4:**
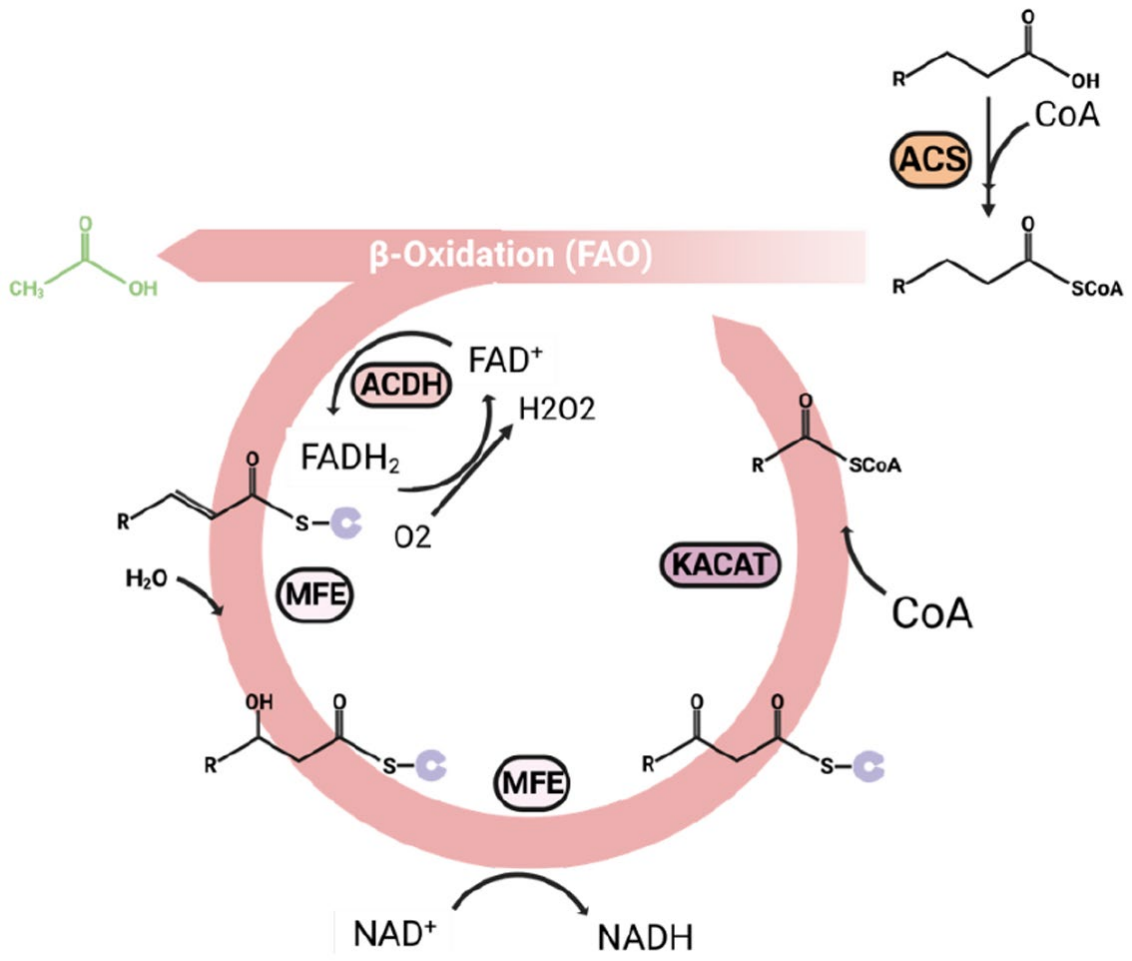
An overview of the beta-oxidation process, which is also a reverse reaction of the fatty acid synthesis. Every beta-oxidation cycle can release an acetyl-CoA and shorten the carbon chain of acyl-CoA by two carbons. ACS, acyl-CoA synthesis; KACAT, β-keto thiolase; MFE, Multifunctional enzyme; ACDH, acyl-CoA dehydrogenase.

As a high-efficiency energy and carbon resource stored in cells, the fatty acids can degrade to acetyl-CoA for energy or carbon utilization (Figure 4). Beta-oxidation is the primary pathway for fatty acid degradation. However, as the final product of omega-3 fatty acids biomanufacturing, beta-oxidation is unfavorable for omega-3 fatty acids accumulation. Consequently, a deeper understanding of beta-oxidation can guide us in designing highly effective metabolic pathways within organisms and optimizing fermentation parameters in biomanufacturing omega-3 fatty acids.

The beta-oxidation mainly happens in the peroxisome. After the fatty acids are activated to form acyl-CoA by acyl-CoA synthesis (ACS), the long-chain acyl-CoA can transfer to peroxisome by ABC transporter, and the short-chain to medium-chain acyl-CoA entering peroxisome by free diffusion (Van Roermund et al., 2012). Three major enzymes inside the peroxisome catalyze the rest of beta-oxidation processing, including acyl-CoA dehydrogenase (ACDH) encoded by POX genes, Multifunctional enzyme (MFE) encoded by MFE genes (Matsuoka et al., 2003), and β-keto thiolase (KACAT) encoded by POT1 gene. The MFE is a big enzyme complex with several domains with different catalyze centers, including enoyl CoA hydratase (ECH) and 3-hydroxyacyl-CoA dehydrogenase (HADH) function. The acyl-CoA can then be degraded in the following four steps:

(1) Dehydrogenated by ACDH generate α, β-enoyl-CoA.
(2) Forming β-hydroxyacyl-CoA by catalyzing by MFE.
(3) Converting β-hydroxyacyl-CoA to β-ketoacyl-CoA by MFE (Wang et al., 2022).
(4) Releasing one molecule of acetyl-CoA and an acyl-CoA with two carons decreased.

The shortened acyl-CoA can continue to go through beta-oxidation processing until it fully converts to acetyl-CoA (Werner and Zibek, 2017).

Most beta-oxidation occurs within the peroxisome, making this organelle crucial for the beta-oxidation process (Tan et al., 2019). The PEX genes encode peroxisomal membrane proteins (PMPs) and are essential for peroxisome formation. It is widely accepted that peroxisomes originate from the endoplasmic reticulum, with the expression of Pex3p and Pex16p proteins encoded by PEX3 and PEX16 inserted into the endoplasmic reticulum, respectively, initiating the formation of peroxisomes. Subsequently, other peroxisomal membrane proteins begin to synthesize and insert into pre-peroxisomes, signifying the maturation of the peroxisome (Dansen et al., 2001). Pex10p, among all other peroxisomal membrane proteins, can significantly influence lipid degradation and synthesis. Xue et al. (2013) report that in the knockout of the PEX10 gene in the omega-3 fatty acids biomanufacturing strain of *Yarrowia lipolytica*, the EPA content nearly doubled, demonstrating that the deletion of PEX10 resulted in a beta-oxidation defect.

### 3.2. Omega-3 synthesis pathway

For omega-3 fatty acid biosynthesis, two major metabolic pathways are considered possible to utilize for omega-3 fatty acid manufacturing: the aerobic fatty acid desaturation pathway and the anaerobic polyketide synthase pathway.

#### 3.2.1. Aerobic unsaturated fatty acids synthesis pathway for omega-3 fatty acid

**Figure 5:**
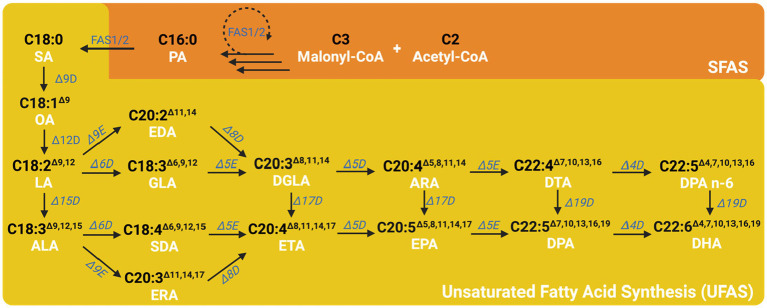
The general metabolic pathway for the synthesis of omega-3 fatty acids. After the saturated fatty acid is generated in the FAS pathway, it is further desaturated by the unsaturated fatty acid synthesis pathway by several different desaturases and elongases. PA, Palmitic acids; SA, Stearic acid; OA, Oleic acid; LA, Linoleic acid; ALA, Alpha-Linolenic acid; GLA, Gamma-Linolenic acid; SDA, Stearidonic acid; DGLA, Dihomo-Gamma-Linolenic acid; ETA, Eicosatetraenoic acid; EDA, Eicosadienoic acid; ERA, Eicosatrienoic acid; ARA, Arachidonic acid; EPA, Eicosapentaenoic acid; DTA, Docosatetraenoic acid; DPA, Docosapentaenoic acid; DHA, Docosahexaenoic acid; Δ9D, Δ9 desaturase; Δ12D, Δ12 desaturase; Δ15D, Δ15 desaturase; Δ6D, Δ6 desaturase; Δ5E, Δ5 elongase; Δ17D, Δ17 desaturase; Δ9E, Δ9 elongase; Δ19D, Δ19 desaturase; Δ4D, Δ4 desaturase.

The aerobic unsaturated fatty acid desaturation/elongation pathway is a conventional metabolic route that involves several different desaturases and elongases of fatty acids for adding double bonds or carbon to different positions or lengths of fatty acids (Figure 5).

After eight cycles of the fatty acid synthesis, stearic acid (SA, C18:0) is transferred into the endoplasmic reticulum and undergoes the first unsaturation step. Stearic acid can be desaturated by Δ9 desaturase, which forms a double bond from the 9th carbon away from the ACP side, resulting in oleic acid (OA, C18:1^Δ9^) (Jónasdóttir, 2019). Subsequently, oleic acid can be further desaturated by Δ12 desaturase at the 12th carbon away from the ACP side, yielding linoleic acid (LA, C18:2^Δ9,12^) (Zhuang et al., 2022). Linoleic acid can be further desaturated by Δ15 desaturase at the 15th carbon away from the ACP side, resulting in alpha-linolenic acid (ALA, C18:3^Δ9,12,15^). Both LA and ALA can be further desaturated by Δ6 desaturase at the 6th carbon away from the ACP side, producing gamma-linolenic acid (GLA, C18:3^Δ6,9,12^) and stearidonic acid (SDA, C18:4^Δ6,9,12,15^), respectively. These compounds can then undergo the addition of an acetyl-CoA to the ACP side, which elongates two carbons on the main carbon chain by Δ5 elongase, resulting in dihomo-gamma-linolenic acid (DGLA, C20:3^Δ8,11,14^) and eicosatetraenoic acid (ETA, C20:4^Δ8,11,14,17^), respectively. Dihomo-gamma-linolenic acid can be desaturated to ETA by Δ17 desaturase. LA and ALA can also enter another pathway where an acetyl-CoA is added to the ACP side to elongate two carbons on the main carbon chain by Δ9 elongase, yielding eicosadienoic acid (EDA, C20:2^Δ11,14^) and eicosatrienoic acid (ERA, C20:2^Δ11,14,17^), respectively. EDA and ERA can be further desaturated by Δ6 desaturase at the 8th carbon away from the ACP side, producing DGLA and ETA, respectively. These compounds can then be further desaturated by Δ5 desaturase at the 5th carbon away from the ACP side, resulting in arachidonic acid (ARA, C20:4^Δ5,8,11,14^) and eicosapentaenoic acid (EPA, C20:5^Δ5,8,11,14,17^), respectively. ARA can be desaturated to EPA by Δ17 desaturase. In following pathway, ARA and EPA can have an acetyl-CoA added to the ACP side to elongate two carbons on the main carbon chain through the action of Δ5 elongase, resulting in docosatetraenoic acid (DTA, C22:4^Δ7,10,13,16^) and docosapentaenoic acid (DPA, C22:5^Δ7,10,13,16,19^), respectively. DTA can then be desaturated to DPA by Δ19 desaturase. Finally, DTA and DPA can be further desaturated by Δ4 desaturase at the 4th carbon away from the ACP side, yielding DPA and docosahexaenoic acid (DHA, C22:6^Δ4,7,10,13,16,19^), respectively. DPA can be desaturated to DHA by Δ19 desaturase. The aerobic pathway advantage has been more extensively researched and understood, simplifying the process of genetic manipulation for fatty acid production. However, it can have a drawback of lower yield and efficiency due to the stepwise and complex nature of the process (Ruiz-Lopez et al., 2015).

#### 3.2.2. Anaerobic polyketide synthase pathway for omega-3 fatty acid

**Figure 6:**
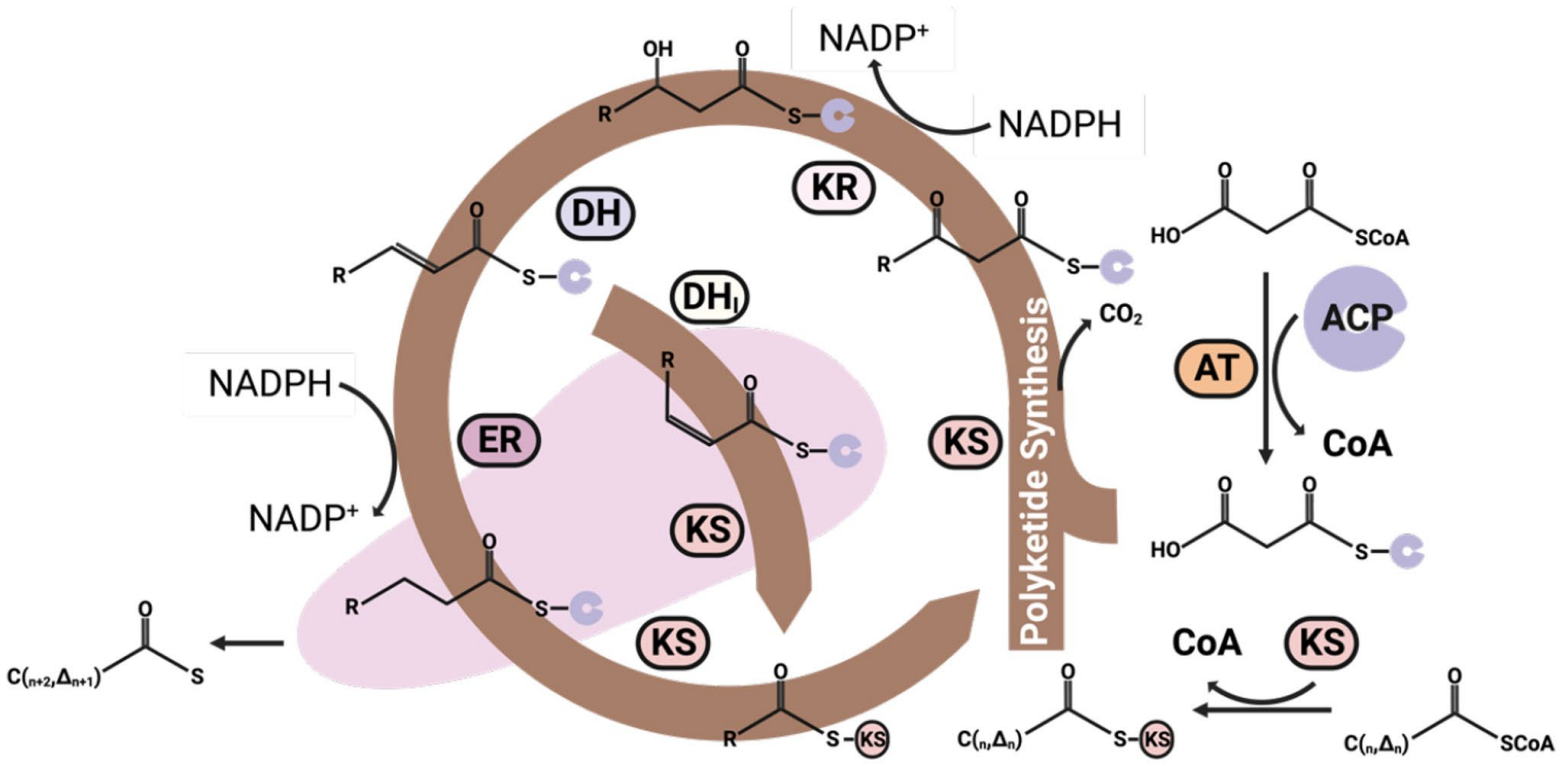
The polyketide synthesis (PKS) pathway for the synthesis of omega-3 fatty acids. The malonyl-CoA can be added to acetyl-CoA every cycle of the PKS pathway to increase two carbons on the Acyl-CoA carbon chain. However, the major difference between the PKS and FAS pathways is that 2,3-trans-enoyl-CoA can isomerize by DH_i_, thus generating a cis double bond during the carbon chain elongate processing without one NADPH consuming. DH_i_, isomerase.

The anaerobic polyketide synthesis pathway can synthesize very long-chain fatty acids, starting directly from acetyl-CoA. This pathway employs a large multifunctional enzyme, polyketide synthase (PKS), with eight different protein domains. These domains work in concert to extend the fatty acyl chain, simultaneously introducing a double bond every two carbons during the extension process (Figure 6; Metz et al., 2001; Hayashi et al., 2016). The primary advantage of the PKS pathway is that it potentially offers a more efficient and direct way to synthesize EPA or DHA, as it condenses the desaturation and elongation processes into a single reaction cycle.

The PKS pathway presents an alternative for synthesizing omega-3 fatty acids from acetyl-CoA in an anaerobic environment (Li Z. et al., 2018). It bears considerable similarity to the fatty acid synthesis pathway, which initiates with acetyl-CoA coupling to KS and subsequent binding with malonyl-CoA to form alpha-ketoacetyl-ACP, concurrently releasing one CO_2_ molecule.

DH then reduces the alpha-ketoacetyl-ACP to yield enoyl-ACP and H_2_O. Enoyl-ACP can undergo further reduction or isomerization to generate an acyl-ACP with an increased chain length by two carbons or an acyl-ACP with an expanded carbon chain and a cis double bond.

This newly formed saturated or unsaturated acyl-ACP can proceed into the lipid synthesis pathway, primarily incorporating into the sn-2 position of LPA (Ma et al., 2022). Alternatively, it may bind with another malonyl-CoA molecule, reentering the PKS synthesis cycle to continue the elongation of the carbon chain and the introduction of additional double bonds. Each cycle of the PKS pathway can add two carbons and one double bond to an acyl-CoA (Gemperlein et al., 2014).

The pathway clearly illustrates that the initial three steps closely parallel the processes involved in the FASs. The primary divergence occurs in the fourth step, where an isomerase facilitates the transformation of the trans double bonds in enoyl-ACP into cis double bonds. PKSs and FASs demonstrate shared evolutionary traits, yet PKSs lack one or more catalytic sites necessary for fatty acid elongation. This results in a longer acyl chain that a keto group, a double bond, or a secondary alcohol group characterizes (Blasio and Balzano, 2021). This unique aspect enables direct bond formation without necessitating the consumption of NADPH for double bond reduction. This strategy also conserves a redox equivalent, providing a significant advantage in the subsequent desaturation stages of the double bonds.

Intriguingly, the PKS pathway notably reduces NADPH requirements for synthesizing EPA and DHA. To illustrate, synthesizing EPA and DHA from malonyl-CoA and acetyl-CoA through the FAS and desaturase/elongase pathways necessitates 21 and 26 units of NADPH, respectively. Specifically, it uses 8 and 12 moles less NADPH than the FASs for forming EPA and DHA, respectively (Jia et al., 2022). NADPH is typically a limiting component in omega-3 fatty acid synthesis, and the enhanced redox efficiency of the PKS pathway makes it a highly competitive alternative for omega-3 fatty acid biomanufacturing (Gemperlein et al., 2019).

However, The PKS pathway involves a complex set of enzymes and requires precise regulation. The exact mechanisms of this pathway are still under investigation, making it a challenging candidate for genetic engineering, which poses a challenge in exploiting this pathway for large-scale production.

#### 3.2.3. FFA and TAG formation and conversion pathways

**Figure 7:**
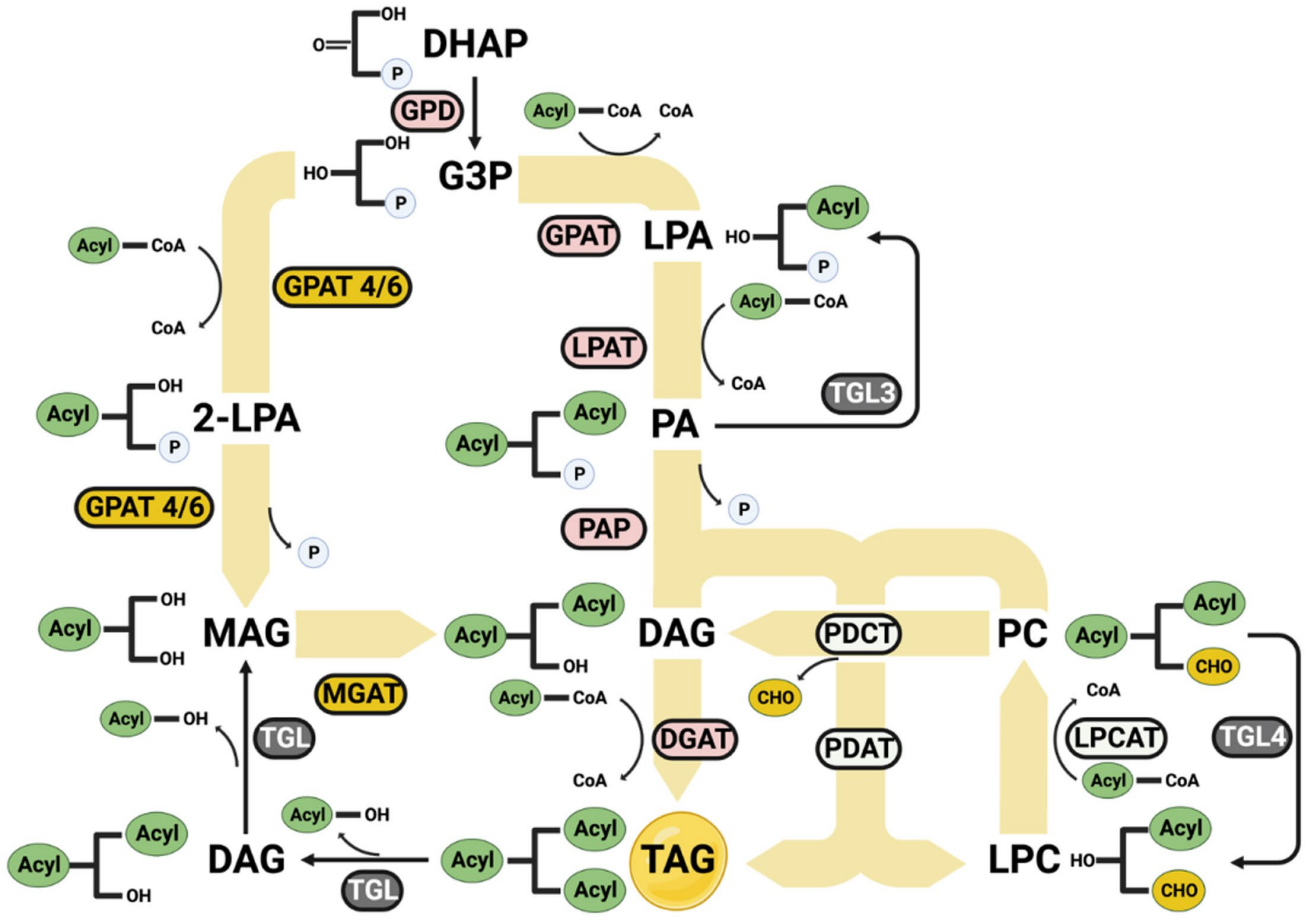
Formation of omega-3 triglycerides. The synthesized omega-3 acyl-CoAs can enter the lipid synthesis process to form a more stable compound, lipid triglyceride. GPD, Glyceraldehyde-3-phosphate dehydrogenase; GPAT, Glycerol-3-phosphate acyltransferase; LPAT, 1-acyl-sn-glycerol-3-phosphate acyltransferase; PAP, 1,2-diacyl-sn-glycerol 3-phosphate phosphohydrolase; DGAT, 1,2-diacyl-sn-glycerol O-acyltransferase; GPAT 4/6, Glycerol-3-phosphate 2-O-acyltransferase 4/6; MGAT, Monoacylglycerol O-acyltransferase; LPCAT, Lysophosphatidylcholine acyltransferase; PDCT, phosphatidylcholine diacylglycerol choline phosphotransferase; PDAT, phospholipid diacylglycerol acyltransferase; LPA, Lysophospholipid; PA, phospholipid; DAG, di-glyceride; PC, phosphatidylcholine; LPC, lysophosphatidylcholine; 2-LPA, 2-lysophospholipid; MAG, Monoglyceride.

The free fatty acid accumulation inside cells is toxic to cells. FFAs can integrate into cell membranes, altering their fluidity, functionality, and integrity, disrupting normal cellular processes (Figure 7). It also can lead to an overload of fatty acid beta-oxidation in the mitochondria. The increase of H2O2 due to excess free fatty acids under the beta-oxidase process leads to the increased production of reactive oxygen species (ROS) (Egnatchik et al., 2014). These ROS can damage cellular structures and lead to cell death. Accumulation of FFAs can induce endoplasmic reticulum (ER) stress, which can also trigger cell death if the stress is too severe or prolonged (Ly et al., 2017).

Thus, it is crucial for omega-3 fatty acids to be stored in an appropriate format. Triglyceride is a generally stable format for storing fatty acids. Once the omega-3 fatty acids are synthesized through the fatty acid synthesis pathway, they attach to a glycerol backbone to form triglycerides under the lipid synthesis pathway (Jiang et al., 2022). The primary lipids synthesis pathway, the Kennedy pathway, happens in the endoplasm reticulum (Arhar et al., 2021). The acyl-CoA can be added to Glycerol-3-phosphate is activated by GPD1 from DHAP by following steps (Klug and Daum, 2014):

(1) An acyl-CoA is added to the sn-1 position of G3P to form lysophospholipid (LPA) by GPAT (Jain et al., 2007).
(2) Another acyl-CoA is added to the sn-2 position of the LPA to form phospholipid (PA) by LPAT.
(3) The phosphate group is removed from the sn-3 position of the PA to form di-glyceride (DAG) by PAP.
(4) The last acyl-CoA is added to the sn-3 position of the DAG to form triglyceride (TAG) by DGAT (Wang et al., 2012).

Other pathways can also add acyl-CoA to the glycerol backbone from TAG:

(1) G3P can add an acyl-CoA to the sn-2 position and generate a 2-lysophospholipid (2-LPA) by GPAT4/6.
(2) The 2-LPA releases the phosphate group to form a Monoglyceride (MAG) by GPAT4/6.
(3) MAG adds another acyl-CoA by MGAT to produce a DAG, thereby entering the Kennedy pathway (Petrie et al., 2012; Radulovic et al., 2013).

Through the phosphatidylcholine synthesis pathway, the phosphatidylcholine (PC) can transfer an acyl group to DAG to form a TAG and a lysophosphatidylcholine (LPC) by PDAT (Benghezal et al., 2007; Batra, 2023). Meanwhile, the LPC can receive an acyl group catalyzed by LPCAT (Chen et al., 2007; Zheng et al., 2012), and the PC can enter the Kennedy pathway by converting to DAG by PDCT.

## 4. Perspectives for future biomanufacturing of omega-3 fatty acids

### 4.1. Increasing NADPH availability such as recycling NADH back to NADPH for lipid synthesis

NADPH is a significant requirement in the *de novo* biosynthesis of omega-3 fatty acids, which require 21 and 26 NADPH units for EPA and DHA biosynthesis, respectively, from acetyl-CoA via the FAS pathway.

Wasylenko et al. (2015) in a metabolic flux analysis, determined that the pentose phosphate (PP) pathway is the primary source of NADPH for lipogenesis. A model developed by Qiao et al. (2017) to analyze the global metabolic network during lipid accumulation found that a limited supply of NADPH restricts lipogenesis. This suggests that lipid accumulation could be enhanced by increasing the supply of NADPH. Qiao et al. engineered four pathways to convert glycolytic NADH into cytosolic NADPH to address this issue. They employed various strategies, including the overexpression of NADP^+^-dependent G3P dehydrogenases, activation of the POM cycle, and activation of the NOG pathway. These strategies significantly increased lipid yield. Co-expression of a heterologous GapC and a heterologous MCE2 further boosted the lipid yield. In an optimized bioreactor, the highest lipid yield reached was 0.279 g/g, with a productivity of 1.2 g/(L·h) and a lipid titer of 99.3 g/L (Wang J. et al., 2020). Therefore, a better design of the NADPH metabolic pathway in fatty acids synthesis is crucial for omega-3 fatty acid biomanufacturing in the future.

### 4.2. The production of omega-3 fatty acids in yeast as an extracellular product has been pursued its potential to yield higher titer

Conventionally producing omega-3 fatty acids *in vivo* entails harvesting and purifying the oil from the cells. This approach imposes a boundary on production potential, as the cell volume limits the maximum yield. The omega-3 fatty acids are synthesized within and then secreted outside the cells, enabling the rate of omega-3 fatty acids over the dry cell weight to exceed 100%. This strategy might not only increase the titer of the omega-3 fatty acids, but it could also decrease the cost of downstream harvesting and processing. Ledesma-Amaro et al. developed two approaches to secrete FA out of cells.

The first approach to this innovative method sought to increase the FFA flux by overexpressing genes involved in TAG synthesis and degradation (*DGA2, TGL4*, and *TGL3*). Concurrently, genes associated with FFA activation and degradation (*FAA1* and *MFE1*) were deleted. The result was an accumulation and subsequent secretion of FFA. The second approach aims to entirely prevent lipid body (LB) formation and redirect FA synthesis to the cytosol by emulating bacterial pathways with relocated acyl-CoA thioesterases. This redirection was achieved by deleting four genes (*ARE1, DGA1, DGA2*, and *LRO1*) that inhibit the formation of neutral lipids (TAG and SE), in combination with FAA1 and MFE1 deletion, and the overexpression of *RnTEII* to enhance FA secretion (Ledesma-Amaro et al., 2016). When these methods were employed in an optimized bioreactor, a fatty acid titer of 10.4 g/L was achieved, yielding 0.20 g/g and a total equivalent lipid content of 120.4% of the dry cell weight (DCW) (Wang J. et al., 2020). This surpassed the storage capacity of individual cells. These groundbreaking strategies disentangle production from biomass formation and simplify product extraction, offering a potential breakthrough for overcoming existing constraints in microbial lipid production. Thus, considering secret the omega-3 fatty acids out of cells for omega-3 fatty acids biomanufacturing have great potential.

### 4.3. New gene editing tools to accelerate the metabolic engineering research

Synthetic biology has dramatically promoted the biological production of omega-3 fatty acids, in which the advancement of molecular biology tools has played a critical role. An increasing number of molecular biology tools are becoming available to aid in implementing designs from metabolic engineering (Bredeweg et al., 2017).

#### 4.3.1. CRISPR gene-editing tools

Gene-editing tools such as CRISPR can precisely insert or delete targeted genes and tune gene expression levels (Liu et al., 2015). CRISPR-Cas technology is one of the most cutting-edge gene editing technologies developed in molecular biology. Its emergence has provided scientists with a precise method for gene editing in model and non-model organisms. It is especially effective for gene editing in non-model species where traditional methods are inefficient. This gene editing technology can not only precisely knock out and insert genes into target species, but it can also utilize its accurate gene targeting function to perform gene editing tasks such as gene activation (CRISPRa) (Bikard et al., 2013), interference (CRISPRi) (Qi et al., 2013), and point mutation (CRISPR-nCas9) (Li X. et al., 2018). This precise gene editing of the target species allows for subtle adjustments of carbon and energy flow (Jeong et al., 2023), balancing the carbon-energy balance within the organism, thereby enhancing the high yield and high-productivity production of omega-3 fatty acids.

#### 4.3.2. Enzyme engineering

Increasing or decreasing the expression of key enzymes in metabolic pathways at the genetic level to drive carbon flow toward product direction and thereby increase product yield. However, for some heterologous enzymes, integrating them into hosts may decrease their catalytic activities or even cause a loss of function. Therefore, improving enzymatic activity is a key goal. Protein engineering can enhance an enzyme’s catalytic abilities, thus increasing the conversion efficiency of a metabolic pathway. Several approaches have been used to achieve this, including using artificial enzymes, direct enzyme evolution, and enzyme immobilization (Pröschel et al., 2015; Mateljak et al., 2019; Li et al., 2020). As part of protein engineering, directly modifying or immobilizing key enzymes to enhance their enzymatic activity, co-localizing enzyme complexes, and improving protein stability is cutting-edge biotechnology that can increase the titer and productivity in ω-3 fatty acids biomanufacturing.

Sellés Vidal et al. (2021) introduce a novel artificial selection method that improves enzymes by connecting their properties to the growth of *E. coli* cells. The process exploits enzymes using cofactors NAD^+^ or NADP^+^ to compensate for faulty NAD^+^ regeneration induced by inactivating specific genes in *E. coli*, causing a conditional growth defect. However, this defect can be remedied by foreign enzymes, provided their substrates are present. The researchers successfully used this principle to isolate beneficial variants of alcohol dehydrogenase, imine reductase, nitroreductase, and a high-performing isopropanol metabolic pathway from large libraries of variants in single-round experiments. This artificial selection approach provides an efficient pathway for developing enhanced enzymes with potentially wide-ranging applications in omega-3 fatty acids bio-manufacturing.

Assembling metabolic pathways with multiple enzymes results in flux imbalance, limiting overall conversion efficiency and accumulating intermediates that can be toxic to host cells. Research has focused on improving pathway efficiency through both the design of artificial enzyme complexes and the spatial arrangement of these complexes (Seo and Schmidt-Dannert, 2021). For instance, a fusion enzyme combining yeast’s Erg20p and Bts1p was created to enhance diterpene production. It effectively channeled key substrates into diterpene products. Combining two such fusion enzymes increased miltiradiene production in a 15 L bioreactor with 365 mg/L (Zhou et al., 2012). Moreover, fusion enzymes have been developed to reduce substrate diffusion, minimize intermediate toxicity, and increase carbon flux. These have been used to improve production in a variety of pathways significantly.

One notable strategy is physically bringing enzymes that catalyze consecutive reactions closer together, using synthetic ‘scaffold’ structures inspired by naturally occurring protein binding domains. This approach has achieved significant increases in production in various pathways. Synthetic nucleic acid scaffolds have also been used to enhance natural product bioconversion, such as using Zif268 and PBSII zinc-finger domains in resveratrol biosynthesis, increasing the tier by three fold (Conrado et al., 2011).

An alternative method for optimizing enzyme assembly is to mimic bacterial microcompartments (BMCs), moving enzymes to a subcellular space. While this technique holds great potential, it is currently hindered by a limited understanding of assembly and functionalization mechanisms and several other challenges (Li et al., 2020). As a multiple-enzyme catalyzed pathway, it has great potential to use all these methods to increase omega-3 fatty acids production.

#### 4.3.3. Multi-omics analyses

Multi-omics analyses, incorporating metabolomics, transcriptomics, and proteomics, can provide a comprehensive understanding of the fungal factory after engineering. Such in-depth knowledge is helpful for subsequent design stages and can be employed for *in silico* calculations using the gathered data.

### 4.4. Using alternative/economical feedstocks to increase yield and reduce material cost

Using other coast-efficiency carbon source, including lipids from waste cooking oil, cellulose and hemicellulose from lignocellulose, starch, sucrose from molasses, other than glucose for omega-3 fatty acids manufacturing have several advantage including decreasing the cost of manufacturing, increase the titer of omega-3 fatty acids, and environment friendly (Ledesma-Amaro and Nicaud, 2016).

For some of these low-cost carbon sources, cells may not utilize them naturally. Thus, engineering the cells to degrade them is the first step to switching from a high-cost carbon source to a low-cost one. Here are examples of *Y. lipolytica* utilization of different carbon sources (See also Table 3).

**Table 3:** Examples of using alternative substrates for lipids and lipid-derived products.

Substrate		Host	Product	Titer/Rate/Yield (TRY)	References
Waste plant oils or animal fats	Waste cooking oil	*Yarrowia lipolytica* (YlB6, YlC7 and YlE1 mutants)	C16 and C18 SFAs and MUFAs	0.062, 0.044, and 0.041 g/L/h	Katre et al. (2017)
	Pork lard	*Yarrowia lipolytica* (W29)	Microbial lipids, citric acid, and lipase	Lipid: 58% (w/w)	Lopes et al. (2018)
	Olive mill wastewater	*Yarrowia lipolytica* (ACA-YC 5033)	Microbials lipids, citric acid, phenolic compound removal	Lipid: 48% of DCW	Sarris et al. (2017)
Cellulose	Cellobiose	*Yarrowia lipolytica* (ZetaB 1 and ZetaB 2)	Microbial lipids	0.8 g/L	Guo et al. (2015a)
	Wheat straw hydrolysate	*Saccharomyces cerevisiae, Lipomyces starkeyi,* and *Rhodotorula babjevae*	Microbial ipids and ethanol	0.08 g/h and 0.18 g/h lipid production rates for *L. starkeyi,* and *R. babjevae,* respectively	Brandenburg et al. (2018)
Starch	Raw starch	*Yarrowia lipolytica* (JMY5196)	Microbial lipids	27% of DCW	Ledesma-Amaro et al. (2015)
	Starch wastewater	*Coculture of anaerobic sludge and microalga Scenedesmus* sp.	Hydrogen and microbial lipids	0.36 g/L	Ren et al. (2015)
	Cassava starch	Coculture *Rhodosporidium toruloides* and amylases-producing yeast *Saccharomycopsis fibuligera*	Single cell oil	64.9% (w/w)	Gen et al. (2014)
Sucrose		Cocoulture of *Chlorella pyrenoidosa* and *Rhodotorula glutinis*	Microbial lipids and citric acid	up to 30% (w/w)	Wang et al. (2016)
C_1_/C_2_ chemicals	Methanol	*Ogataea polymorpha*	Free fatty acids	15.9 g/L	Gao et al. (2022)
		*Pichia pastoris*	Free fatty acids	23.4 g/L	Cai et al. (2022)
	CO_2_	*Chlorococcum littorale*	Free fatty acids	47.8 wt % of DCW	Ota et al. (2015)
	CO_2_	*Chlorella vulgaris*	Microbial lipids	38 ± 2.6% of DCW	Jain et al. (2019)
	CO_2_	*Haematococcus pluvialis*	Omega-3 fatty acids and astaxanthin	171 mg/L	Yu et al. (2021)
	Acetic acid obtained from syngas	*Moorella thermoacetica* and *Yarrowia lipolytica*	C16-C18 TAGs	18 g/L	Hu et al. (2016)
	Acetic acid	*Rhodosporidium toruloides*	Microbial lipids	48.2% of DCW	Huang et al. (2016)
	Acetic acid in both salt and acid forms	*Yarrowia lipolytica*	TAG	115 g/L	Xu et al. (2017)
	Acetate	*Cryptococcus curvatus*	Microbial lipids	73.4% of DCW	Gong et al. (2015)

#### 4.4.1. Waste plant oils/animal fats

Based on the native *Y. lipolytica* theoretically yields show that it needs 3.69 kg glucose to synthesize 1 kg stearic acids (C18:0) (Qiao et al., 2017), then the stearic acids be further used for EPA and DHA synthesis. Directly using lipids as the carbon source for omega-3 synthesis is much more efficient for the omega-3 synthesis (Soong et al., 2023). The oil price is only twice that of glucose, but oil synthesis needs almost four times that of glucose. As a result, using oil as a feedstock for omega-3 synthesis may help cut the total cost by nearly half compared to using glucose.

#### 4.4.2. Cellulose

Cellulose, a linear polysaccharide comprising glucose subunits, requires at least three classes of cellulases to break it down: endoglucanases, cellobiohydrolases, and beta-glucosidases. Through the overexpression of endogenous or heterologous beta-glucosidases, *Y. lipolytica* can grow in cellobiose (Guo et al., 2015b). Strains were engineered to express one of three genes: *Trichoderma reesei EGII*, *T. reesei CBHII*, or a *T. reesei–Talaromyces emerso*nii chimeric *CBHI*. The most successful growth and cellulose consumption occurred when all three strains were combined, though the conversion efficiency remains low at 23%, indicating room for improvement (Wei et al., 2014).

#### 4.4.3. Starch

Despite being an abundant carbohydrate, *Y. lipolytica* cannot naturally metabolize starch due to its lack of alpha-amylase and glucoamylase. A strain has been engineered to express and secrete both enzymes to address this. This advancement allowed it to grow on soluble starch (post-liquefaction) and raw starch. The genetic construct with these two genes was introduced into a genetically modified strain that accumulated large amounts of lipids, enabling biodiesel production from starch (Ledesma-Amaro et al., 2015).

#### 4.4.4. Sucrose

*Yarrowia lipolytica* cannot utilize sucrose as it lacks an invertase-encoding gene. However, researchers have manipulated *Suc* + strains to express the *SUC2* gene from *S. cerevisiae*. *SUC2* has been expressed under the control of the promoter and secretion signal of alkaline extracellular protease (*XPR2*), enabling it as a selection marker in genetic engineering (Fukuda, 2013). Additionally, *SUC2* expression has been employed to produce citric acid from sucrose. The strongest expression has been achieved using the strong TEF promoter, resulting in strains displaying 7.5 times more invertase activity than previous strains (Lazar et al., 2013).

#### 4.4.5. CO_2_-derived C_1_/C_2_ chemicals

**Figure 8:**
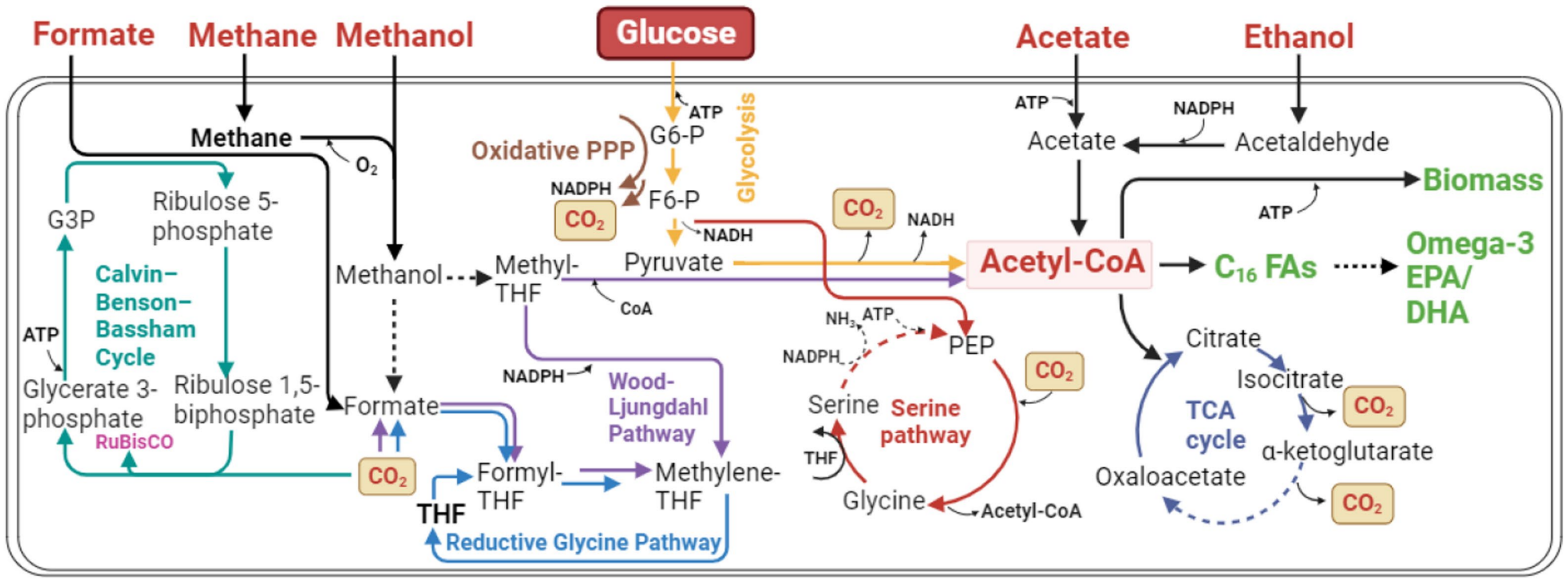
Potential metabolic pathways for using C_1_/C_2_ chemicals for biosynthesis of lipids and omega-3 fatty acids. THF, Tetrahydrofolate; G3P, glyceraldehyde 3-phosphate; G6-P, glucose-6-phosphate; F6-P, fructose 6-phosphate; PEP, phosphoenolpyruvate; RuBisCo, ribulose-1,5-bisphosphate carboxylase. Multi-step reactions are presented by dashed arrows.

C_1_/C_2_ chemicals such as formic acid, methanol, acetic acid, and ethanol can be obtained from the CO_2_ fixation process via an electrochemical catalysis process. This will not only help improve the lipid and omega-3 fatty acid biosynthesis yield but also help address the issues of CO_2_ capturing and fixation related to climate change and the sustainability of biomanufacturing (Xu et al., 2017; Yishai et al., 2018; Zhong et al., 2020). In many fermentation processes, only a small portion of the carbon source is used for biosynthesis of the target product(s), about half or more portion of the carbon source is wasted as off-gas CO_2_ generation (Xu et al., 2021). The released CO_2_ from the fermentation can be fixed into C_1_/C_2_ chemicals via different electrochemical, photochemical, and catalytic processes (Mustafa et al., 2020). In that case, the C_1_/C_2_ chemicals can be brought back to fermentation to significantly improve the overall biomanufacturing yield (Figure 8). Many microbial cells, including methylotrophs, industrial strains, and yeasts, have succeeded considerably in this field.

C_1_ chemicals are deemed as potential feedstocks for bioproduction due to their ubiquitous nature and cost-effectiveness. This interest is further motivated by the urgency to alleviate global warming and reduce dependency on fossil fuels (Zhang et al., 2018; Liu Z. et al., 2020). Nonetheless, using these C_1_ compounds in biomanufacturing poses challenges, including low utilization pathway efficiency, high energy demands, and reduced power (Cotton et al., 2018). Several efforts are underway to optimize these utilization pathways. Microorganisms, such as yeasts and microalgae, have been engineered to utilize these C_1_ substrates effectively. Methylotrophic organisms, capable of using C_1_ compounds as their sole carbon source, are pioneering this advancement. One instance is the engineering of *Ogataea polymorpha*, an industrial yeast, to produce free fatty acids solely from methanol. This process initially had adverse effects, including inhibition of cell growth and potentially cell death. However, through adaptive laboratory evolution (ALE) and the modification of gluconeogenesis pathways, a strain capable of synthesizing fatty acids from methanol was created, achieving a substantial titer of 15.9 g/L (Gao et al., 2022). Similarly, researchers used the methanol utilization pathway of the natural methylotrophic yeast, *Pichia pastoris*, to increase fatty acid synthesis (Peña et al., 2018). The supply of acetyl-CoA was boosted, and NADPH regeneration was intensified to fulfill the large requirement for these components in fatty acid synthesis, which can be directed toward the omega-3 fatty acid production through further genetic modifications. Formaldehyde assimilation was further strengthened, resulting in a strain that could synthesize 23.4 g/L of fatty acids from methanol (Cai et al., 2022). Under methanol cultivation, the cells of Pichia pastoris amplify their peroxisomes, thus exhibiting potential as a host for producing oleochemicals, like lipids and omega-3 fatty acids (Zhou et al., 2016). Moreover, this yeast can be converted into an autotrophic strain, utilizing CO_2_ as a carbon source and methanol as an energy source. This is achieved by engineering the methanol assimilation pathway, known as the xylose monophosphate (XuMp) or dihydroxyacetone (DHA) cycle, into a CO_2_ fixation pathway (Gassler et al., 2020). The thermotolerant methylotrophic yeast, *Hansenula polymorpha*, due to its effective CRISPR-Cas9 mediated genome editing toolkit, presents another promising host for oleochemical production using methanol (Wang et al., 2018). To enhance methanol utilization in other yeasts like *S. cerevisiae* and *Yarrowia lipolytica*, the XuMP cycle from *P. pastoris* has been incorporated into it, leading to slow but progressive growth and pyruvate production (Dai et al., 2017). However, the poor cell growth and slow methanol utilization highlight the need for more sophisticated engineering (Vartiainen et al., 2019).

The utilization of C_1_ compounds is not limited to yeasts. Microalgae are recognized as a significant biofuel feedstock, as these photosynthetic organisms can transform CO_2_ into carbon-rich lipids. Certain CO_2_-tolerant algae, such as *Chlorococcum littorale*, had intracellular fatty acid up to 47.8% DCW using low concentration CO2 (5% v/v) as a carbon source and light as reducing power (Ota et al., 2015). Another microalga, *Chlorella vulgaris*, can directly fix CO_2_; however, fatty acid production is low due to the low solubility of CO_2_ even though the CO2 concentration fed was high up to 20% at a continuous flow rate of 0.5 vvm (Jain et al., 2019). To resolve this, constructing a carbonic anhydrase complex increased CO_2_ solubility by rapidly converting it into bicarbonate (HCO3−), enhancing malonyl-CoA synthesis capacity and ultimately increasing fatty acid production (You et al., 2020). Freshwater microalga, *Haematococcus pluvialis*, already commonly used for industrial astaxanthin production, was proposed to produce omega-3 fatty acids and astaxanthin simultaneously by fixing CO_2_ in calcium-supplemented media (Yu et al., 2021).

Despite the potential, the commercialization of biofuel production from CO_2_ through algal biomass is facing economic hurdles. The cost of producing algal oils and biodiesel from CO2 ranges from 9 to 40 US$/per gallon, making it economically challenging (Salehizadeh et al., 2020). However, with technological advances in cell properties, bioreactor design, and nutrient and energy use, a tenfold reduction in production cost and significant scale-up can be achieved in the coming decade (Khan et al., 2018).

Applying two-carbon (C_2_) chemicals like acetate and ethanol to produce lipids and omega-3 fatty acids has also drawn significant attention in biomanufacturing (Kim et al., 2021). Since ethanol assimilation has not been explored for lipid and/or lipid-derived products, studies focused mainly on acetate. Acetate serves as a precursor for the biosynthesis of crucial biomolecules like amino acids, ketoacids, polyphenols, and fatty acids (Kiefer et al., 2021). Additionally, it is an appealing substrate for this purpose due to its abundance from low-cost sources such as anaerobic digestion and syngas (Hu et al., 2016). However, challenges arise from the low acetate concentration when sourced from upstream waste utilization processes. This issue has been addressed by developing advanced feed strategies and process setups, such as continuous bioreactors and cell recycling units (Xu et al., 2017).

Microorganisms are crucial in these processes, with various natural hosts and industrial strains being studied. The oleaginous yeasts *Rhodosporidium toruloides*, *Yarrowia lipolytica*, and *Trichosporon cutaneum* are particularly interesting. These yeasts have been investigated for lipid production solely from acetate (Zhang C. et al., 2022). For instance, an engineered strain of *Y. lipolytica* grown on 30% (v/v) acetic acid yielded a lipid titer of 51 g/L and lipid accumulation of 61%. The yeast’s efficacy was further demonstrated by applying a cell recycling scheme using 3% (v/v) acetic acid media. This led to a lipid titer of 46 g/L and a lipid accumulation of 59% (Hu et al., 2016). Integrating multi-step bioprocesses and microbial consortia can drastically increase productivity and reduce costs. A two-step biosynthetic system that combines coculture systems with industrial and nonconventional strains may also have promising results (Du et al., 2020). Under optimized feeding conditions and combining this with a semi-continuous process, the previous result has improved to accumulate triacyl glycerides (TAGs), intracellular products in the bioreactor, more than twofold over the earlier results, achieving a TAG titer of 115 g/L and a TAG productivity of 0.8 g/L/h (Xu et al., 2017). This means the effect of using different organisms is broader than individual cultures.

However, the use of acetate as a sole carbon source poses several challenges, including its low concentration when produced from many upstream waste utilization processes and the necessity of deriving NADPH from the pentose phosphate pathway (PPP), which requires a long pathway to be connected to acetate. Solutions to these challenges have been explored, such as using a continuous bioreactor with a cell recycling unit to maximize productivity and minimize the effluent’s acetate concentration and the development of a synergistic substrate co-feeding strategy providing gluconate as a secondary substrate. This method enhances acetate-driven lipogenesis via obligatory NADPH synthesis through the PPP without causing carbon catabolite repression that would inhibit acetate assimilation (Park et al., 2019). Another challenge is that acetate metabolism is ATP-costly due to the proton export requirement to prevent acidification when acetate is used in acetic acid form, and this acetic acid is deprotonated at neutral cytosol (Kiefer et al., 2021). Various industrial strains, such as *Cryptococcus curvatus*, have demonstrated promising results in lipid production by using acetate as a carbon source. In batch cultures with 30 g/L acetate, this strain showed 73.4% lipid accumulation and a titer of 4.2 g/L. When grown with corn stover hydrolysate containing multiple substrates, it accumulated a lipid titer of 9 g/L while metabolizing glucose and acetate simultaneously, showing a potential for further improvements with increased access to genetic engineering tools (Gong et al., 2015).

The manipulation of environmental factors such as medium pH, oxygen level, and temperature control also contributes to the success of microbial fermentation. For example, increasing the medium pH from 6 to 8–9 drastically improves cell biomass growth and metabolite production, allowing high acetate loading and yielding much higher cell mass and lipid titers (Gao et al., 2020). At the same time, an optimal temperature is crucial for the fermentation of methanol and ethanol due to the significant heat they generate (Zhang C. et al., 2022).

In summary, significant advancements have been made in C_1_ and C_2_-biomanufacturing, particularly in producing lipids and omega-3 fatty. The roles of natural hosts, environmental parameters, system integration, and genetic engineering tools are pivotal in overcoming challenges and maximizing productivity. Further developing and refining these strategies could lead to an even more efficient and cost-effective bio-production process as we move forward. Despite the challenges, the industrial utilization of C_1_ and C_2_ chemicals for lipid and fatty acid production continues to evolve, offering promising sustainable solutions.

### 4.5. Innovative fermentation and bioprocess engineering.

**Figure 9:**
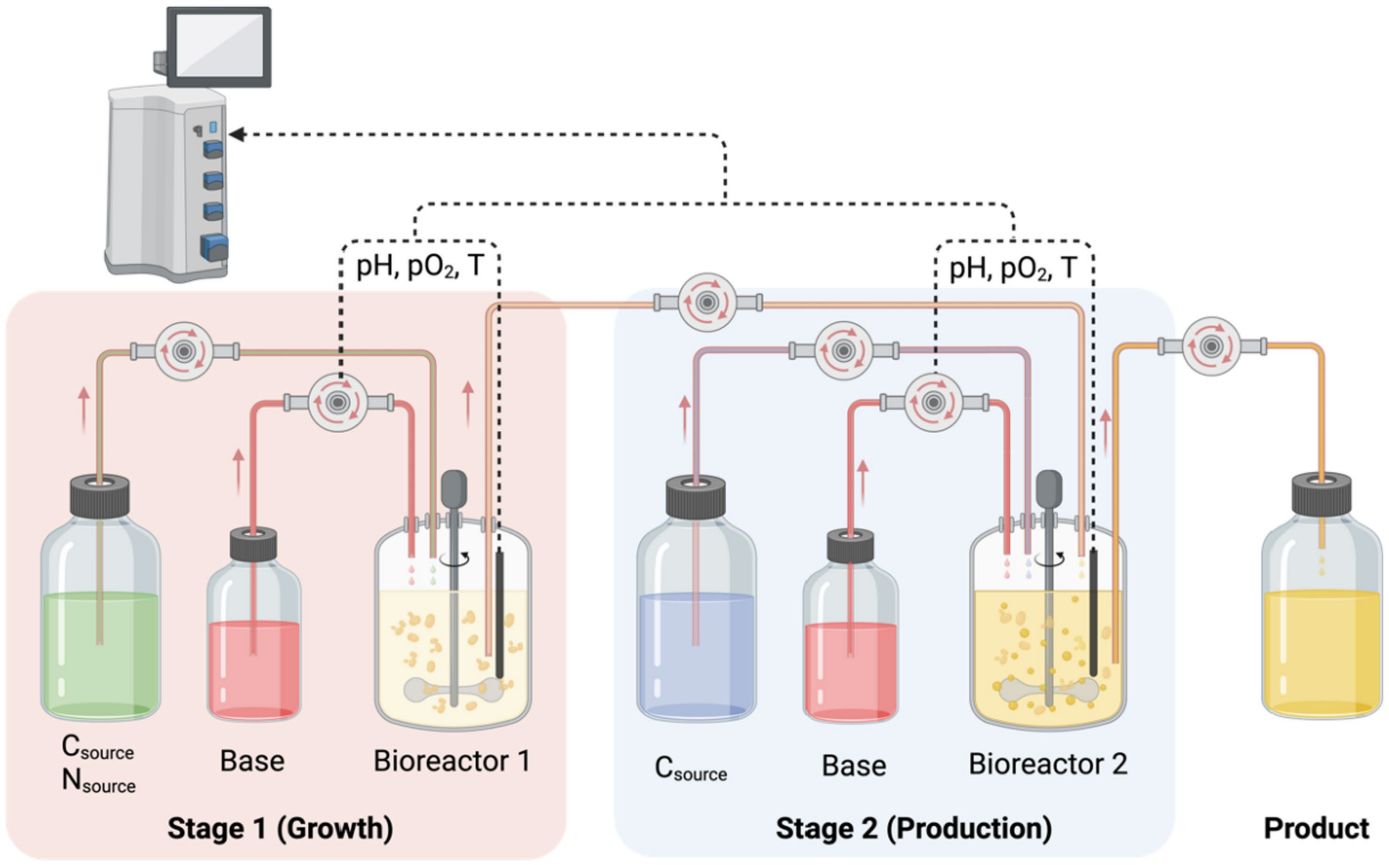
A schematic diagram for a two-stage continuous fermentation process that decouples the growth and production phases. In stage 1 (for nitrogen-rich cell growth), sufficient nitrogen and carbon sources are provided to the bioreactor to support for fast cell growth. In stage 2 (nitrogen-limited for product formation), the bioreactor receives the high-cell-density broth from stage 1 and provides only a carbon source for product formation. The nitrogen-limited environment induces the production of omega-3 fatty acids without further cell growth. Each stage is maintained at its own optimal pH, pO2 (partial pressure of oxygen), and T (Temperature) values during the continuous operation.

The continuous quest for efficient and sustainable lipid production has driven researchers to delve deeply into microbial capabilities. Triglycerides in many microbes serve as energy storage, mainly when nitrogen is scarce, facilitating their survival under harsh conditions. Interestingly, this nitrogen limitation of oleaginous microbes becomes a pivotal factor in producing omega-3 fatty acids. The two-stage continuous fermentation process can be a solution to balance cell growth and lipid accumulation (Jovanovic et al., 2021). However, for these microbes’ production of omega-3 fatty acids, the omega-3 fatty acids are stored in the lipid body inside the cells and require enough nitrogen to grow to a high cell density. Consequently, more supplies of nitrogen sources can be necessary for both the titer and productivity of omega-3 fatty acids. The two-stage continuous fermentation process is a method that can address this issue (Figure 9).

In a two-stage continuous fermentation, cell growth is promoted in the first bioreactor with a sufficient nitrogen supply during the first stage. When the cell density reaches an optimal level in this bioreactor, the cells are transferred to a second bioreactor with a limited nitrogen supply. A minimal nitrogen supply environment triggers the cells to begin accumulating lipids. This strategy balances maintaining adequate cell density and creating a nitrogen-limited environment beneficial for omega-3 production (Xie, 2017). For instance, an engineered *Yarrowia lipolytica* for EPA production was used in a two-stage continuous fermentation, resulting in 25% EPA in the yeast biomass. It was demonstrated that the two-stage process increased overall EPA productivity by 80% and EPA concentration by 40% while maintaining similar conversion yields from glucose compared to the standard fed-batch process (Xie et al., 2017a).

However, there are still some challenges in continuous biomanufacturing. Ensuring a contamination-free environment consistently poses a formidable challenge for extended continuous fermentation processes that span several weeks or even months. Over such lengthy fermentation durations, there is a potential for cells to adapt in ways that promote quicker growth but at the expense of reduced product output, often due to factors like mutations or the loss of plasmids. A notable observation from previous and ongoing continuous fermentation methods is the concurrent struggle to achieve high product titer, rate/productivity, and yield (TRY). This is primarily attributed to the intertwined nature of cell growth and product generation in single-stage continuous fermentation, despite each ideally demanding distinct optimal conditions for medium and processing. Moreover, the research endeavors associated with continuous fermentation tend to be quite labor-intensive and drawn-out. Achieving a stable state can be protracted, necessitating persistent daily oversight of the culture. The field needs more refined research techniques to expedite these continuous processes’ development. Lastly, there needs to be more alignment between the generation and retrieval of products in continuous fermentation. The pivotal nature of downstream recovery in these processes needs to be emphasized. Therefore, there is a pressing need for product extraction methodologies synergized with continuous fermentation and adept at handling flow and concentration variances (Xie, 2022).

Synthetic biology technologies have advanced to track continuous fermentation and provide the optimum media contents for maximizing the product TRY, with numerous tools available for creating microbial cell factories. While these factories can be intricate, synthetic organisms might display unwanted characteristics (Check Hayden, 2015). Thus, computational strain design and high-throughput methods are used to screen these organisms. Techniques such as omics analyses and 13C-metabolic flux analysis help map and measure cellular processes, forming a cycle of design–build–test–learn (DBTL) (Liu et al., 2016; Wu G. et al., 2016). Machine learning (ML) offers an alternative, data-driven approach (Lawson et al., 2021). ML is adept at integrating features ranging from minute elements to larger-scale factors. This capability has shown great potential across numerous crucial aspects of systems metabolic engineering (Volk et al., 2020). These include predicting protein functions and their physiochemical characteristics (Ryu et al., 2019), designing promotors (Wang Y. et al., 2020), designing synthetic pathways (Jang et al., 2022), reconstructing and optimizing metabolic pathways (Kim et al., 2020; Zhang et al., 2020), and applying reinforcement learning to oversee fermentation (Treloar et al., 2020).

Optimization of the fermentation media takes the first place of designing the whole process. It requires conducting a plethora of experiments regardless of the chosen medium. This process is labor-intensive and needs a defined conclusion. Interestingly, data from shake flask experiments often need to align better with fermenter-based studies. Four main shortcomings present in shake flask studies: uncontrollable pH levels, limited oxygen transfer rate, insufficient mixing, and notable evaporation. Commonly, it is assumed that a media performing well in shake flask culture will similarly excel in the fermenter. Furthermore, these media face various challenges on an industrial level, including inconsistencies between batches, year-round availability issues, price fluctuations, transportation stability concerns, and problems tied to bulk storage and duration. However, an in-depth comparing medium performance across different scales could be more extensive (Singh et al., 2017).

For the fermentation process, optimization is also essential. Different ML techniques have been employed to manage fermentation parameters such as temperature, pH, dissolved oxygen, aeration rate, feeding rate, and agitation speed. For instance, Gaussian process regression (GPR) was implemented to forecast the biomass concentration of *S. cerevisiae* based solely on substrate flow rate, given that certain variables could not be measured in real-time (Masampally et al., 2018). Similarly, the challenge of optimum growth temperature (OGT) detection in non-conventional microorganisms has been solved using the amino acid composition as input for employing six different ML models since amino acid composition is strongly related to the OGT (Li et al., 2019).

For the downstream processes, biomass harvesting is one of the most critical steps constituting 20–30% of total production costs (Rashid et al., 2014). Its main challenge is the efficient separation of low-rate and small-sized cells (Nitsos et al., 2020). The goal is to produce a slurry suitable for lipid extraction and, if feasible, recycling of water and nutrients. Centrifugation, flocculation, filtration, and flotation are among the primary techniques utilized for harvesting, and combinations of these methods are also frequent (Sakarika and Kornaros, 2019). Besides, Yin et al. (2020) recently conducted a study collating a variety of flocculants used across multiple microalgae species in the harvesting phase.

Another strategy is employing Adaptive Laboratory Evolution (ALE) to enhance microbial oil. By continuously cultivating cells under specific conditions, ALE enhances its capacity to process custom feedstocks or generate desired metabolites (Arora et al., 2020). It has been particularly useful in boosting the production of commercially significant lipids in oleaginous microbes (Wang X. et al., 2019) —for instance, *Tisochrysis lutea* and *Schizochytrium* sp. HX-308 saw lipid DHA level improvements through one-factor and two-factor ALE, respectively (Sun et al., 2018; Gachelin et al., 2021). Despite its potential, ALE is time-consuming, yet its efficiency can be heightened when paired with tools like high-throughput screening. Notably, strains developed via ALE may revert to their original metabolic profiles, prompting cryogenic storage to prevent such reversions (Uprety et al., 2022). In a broader context, microbial lipids currently face economic challenges in competing with traditional lipid sources, mainly due to the low productivity of oleaginous microorganisms and associated high costs. Presently, only premium Single Cell Oils (SCOs) are industrially produced, even as agro-industrial waste has been explored as a substrate to cut expenses (Bellou et al., 2016). Consequently, there is a push toward optimizing these microorganisms’ lipogenic capabilities, primarily via genetic engineering. Nonetheless, ALE emerges as a promising, cost-effective method to enhance oleagenicity, either as a standalone approach or in tandem with genetic engineering, with significant research undertaken using both heterotrophic and autotrophic microorganisms (Mavrommati et al., 2022).

Cells and microbes are inherently dynamic, possessing numerous internal control mechanisms. However, many optimization studies either oversimplify these organisms, treating them as data-generating entities or relying exclusively on empirical data. Future media optimization strategies should incorporate insights from metabolic pathway regulatory systems (Liu Y. et al., 2020). Furthermore, it is essential to consider the mutation rates in specific media influenced by media components. It should be assumed that such mutations affect the yield or quality of the microbial lipids, potentially paving the way for innovative processes using cost-effective media.

## 5. Conclusion

The crucial role of omega-3 fatty acids as a primary source of pharmaceutical and nutritional components is indisputable, pointing to their significant market potential. Utilizing various microbes as cell factories to produce these essential fatty acids was a method with room for enhancement. Optimizing the biosynthesis of omega-3 fatty acids by increasing carbon yield, balancing cofactors, and reducing by-product formation can increase the product quality of omega-3 fatty acids and lower the cost of biomanufacturing processing. Investigating alternative carbon sources, such as affordable sugars, waste oils/fats, and C_1_/C_2_ chemicals derived from CO_2_, provides an opportunity for additional product yield improvement and cost reduction. Utilizing advanced molecular biology tools can further refine metabolic engineering, increasing the yield of biomanufacturing omega-3 fatty acids from upstream. Moreover, continuous fermentation, AI-controlled fermentation, and other cutting-edge technologies can improve downstream process engineering, elevate productivity, and reduce operational and capital expenses. Collectively, these integrated strategies can serve as a roadmap for elevating the production of omega-3 fatty acids in a manner that is efficient, cost-effective, and sustainable.

## Author contributions

JQ: Conceptualization, Data curation, Formal analysis, Investigation, Methodology, Writing – original draft, Writing – review & editing. EK: Conceptualization, Investigation, Writing – original draft, Writing – review & editing. TL: Investigation, Writing – review & editing. LS: Writing – review & editing. DX: Conceptualization, Funding acquisition, Investigation, Project administration, Supervision, Writing – review & editing.
